# Autologous Versus Allogeneic Adipose-Derived Mesenchymal Stem Cell Therapy for Knee Osteoarthritis: A Systematic Review, Pairwise and Network Meta-Analysis of Randomized Controlled Trials

**DOI:** 10.7759/cureus.82713

**Published:** 2025-04-21

**Authors:** Alousious Kasagga, Anushka Verma, Eiman Saraya, Mehjabin S Haque, Safiyyah M Khan, Pousette F Hamid

**Affiliations:** 1 Pathology, Peking University, Beijing, CHN; 2 Internal Medicine, Smt. Nathiba Hargovandas Lakhmichand (NHL) Municipal Medical College, Ahmedabad, IND; 3 Psychiatry, St. Martinus University, Willemstad, CUW; 4 Internal Medicine, Jiangxi University of Chinese Medicine, Nanchang, CHN; 5 Medicine, Basaveshwara Medical College & Hospital, Chitradurga, IND; 6 Neurology, Faculty of Medicine, Ain Shams University, Cairo, EGY

**Keywords:** adipose-derived stem cells, allogeneic mscs, autologous mscs, functional improvement, knee osteoarthritis, mesenchymal stem cells, network meta-analysis, pain relief, regenerative medicine

## Abstract

Knee osteoarthritis (OA) is a degenerative joint disorder with limited non-surgical treatment options. Adipose-derived mesenchymal stem cell (AD-MSC) therapy has emerged as a promising regenerative approach; however, the comparative efficacy and safety of autologous versus allogeneic AD-MSCs remain unclear. This systematic review and network meta-analysis (NMA) evaluated the effectiveness and safety of intra-articular AD-MSCs in adults with Kellgren-Lawrence Grade II-IV knee OA. A comprehensive search identified eight randomized controlled trials that compared high-dose autologous, high-dose allogeneic, and low-dose allogeneic AD-MSCs to placebo or standard care interventions, such as hyaluronic acid, corticosteroids, or physical therapy. The primary outcomes were pain relief, assessed by the Visual Analog Scale (VAS), and functional improvement, measured by the Western Ontario and McMaster Universities Osteoarthritis Index (WOMAC), at three, six, and 12 months. Treatment rankings were determined using Surface Under the Cumulative Ranking (SUCRA) probabilities. High-dose autologous AD-MSCs ranked highest for pain relief at three, six, and 12 months (VAS SUCRA: 75.99%, 82.27%, 81.65%), while high-dose allogeneic AD-MSCs ranked highest for functional improvement at six and 12 months (WOMAC SUCRA: 74.6%, 71.71%). Low-dose allogeneic AD-MSCs consistently ranked lowest for both outcomes. Adverse event analysis indicated that low-dose allogeneic AD-MSCs had the highest risk of adverse effects (SUCRA: 22.24%), followed by high-dose allogeneic AD-MSCs (26.52%). In contrast, high-dose autologous AD-MSCs ranked safer (SUCRA: 54.08%). Serious adverse events were rare and unrelated to treatment, and consistency testing confirmed no significant inconsistencies in the NMA framework. Overall, high-dose autologous AD-MSCs provided sustained pain relief over 12 months, while high-dose allogeneic AD-MSCs demonstrated superior long-term functional improvement. These findings support a two-phase treatment model in which autologous AD-MSCs offer early and prolonged symptom relief, and allogeneic AD-MSCs assist in long-term joint recovery. Overall, AD-MSC therapy was well tolerated and may represent a viable, personalized, non-surgical knee OA management strategy.

## Introduction and background

Knee osteoarthritis (OA) is a major contributor to pain and disability globally, characterized by progressive cartilage degeneration, chronic synovial inflammation, and structural joint deterioration. Current non-surgical treatments, including nonsteroidal anti-inflammatory drugs (NSAIDs), intra-articular corticosteroids, and hyaluronic acid injections, offer symptomatic relief but do not halt disease progression. With limited therapeutic options short of surgical intervention, mesenchymal stem cell (MSC) therapy has emerged as a promising regenerative approach. MSCs exert anti-inflammatory effects through IL-10 and TGF-β secretion, modulate inflammation, promote tissue repair, and improve joint function [1,2].

Among MSC sources, adipose-derived mesenchymal stem cells (AD-MSCs) have gained significant interest due to their high yield, ease of extraction, and strong proliferative potential. Compared to bone marrow-derived MSCs (BM-MSCs), AD-MSCs can be harvested through minimally invasive liposuction and demonstrate potent paracrine activity, facilitating cartilage repair and immune modulation [3,4]. However, despite increasing clinical investigations, several key uncertainties remain, including the comparative efficacy of autologous vs. allogeneic AD-MSCs, the optimal dosing strategy, and long-term safety considerations [5].

Autologous vs. allogeneic AD-MSC therapy: mechanisms and clinical potential

AD-MSCs exert their therapeutic effects primarily through paracrine signaling, secreting cytokines, growth factors, and extracellular vesicles that modulate immune responses, reduce inflammation, and promote extracellular matrix remodeling. These properties are particularly beneficial in OA, where chronic inflammation accelerates cartilage degradation [6].

AD-MSCs can be autologous (from the patient’s own tissue) or allogeneic (from a donor). Autologous AD-MSCs are considered advantageous due to their immune compatibility, reduced risk of rejection, and potentially longer persistence in the joint environment. Conversely, allogeneic AD-MSCs offer an off-the-shelf treatment option with potentially stronger immunomodulatory effects but may carry a risk of immune recognition and clearance. Some studies suggest that autologous AD-MSCs provide more rapid pain relief, whereas allogeneic AD-MSCs contribute to sustained regenerative effects. Emerging evidence also suggests that therapeutic efficacy may be dose-dependent, with higher cell doses potentially yielding stronger and longer-lasting effects [7]. However, optimal dosing thresholds remain unclear and are rarely compared across studies.

Need for a comprehensive comparative analysis

Despite increasing randomized controlled trials (RCTs) evaluating AD-MSC therapy, variability in study designs, treatment protocols, follow-up durations, and outcome measures has led to conflicting conclusions [8]. While prior meta-analyses have evaluated MSC therapy for OA, none have fully addressed the comparative effectiveness of autologous versus allogeneic AD-MSCs across different doses. Our study addresses this gap using a Bayesian network meta-analysis (NMA), which provides a powerful solution by integrating direct and indirect evidence to enable the comparison of multiple AD-MSC treatment regimens within a unified analytical framework to rank treatment strategies. By evaluating outcomes across multiple time points, we aim to clarify the most effective and safest AD-MSC regimens for knee OA management, with implications for clinical decision-making and trial design.

Study objectives

This systematic review, pairwise and network meta-analysis, aims to comprehensively evaluate the comparative effectiveness and safety of low-dose allogeneic, high-dose allogeneic, and high-dose autologous AD-MSC therapy for knee OA. The primary outcomes include pain relief, measured by the Visual Analog Scale (VAS), and functional improvement, assessed using the Western Ontario and McMaster Universities Osteoarthritis Index (WOMAC) at three, six, and 12 months. Additionally, the study examines the adverse effect (AE) profile of different AD-MSC regimens to identify the most effective intervention based on Surface Under the Cumulative Ranking (SUCRA) probabilities. To ensure the reliability of findings, we perform consistency testing to assess the validity of direct and indirect comparisons within the NMA framework.

Hypothesis and Study Significance

This study hypothesizes that high-dose autologous AD-MSCs will provide rapid pain relief due to their more potent early anti-inflammatory effects. In contrast, high-dose allogeneic AD-MSCs will demonstrate superior long-term functional improvement due to prolonged immune modulation and regenerative activity.

**Figure 1:**
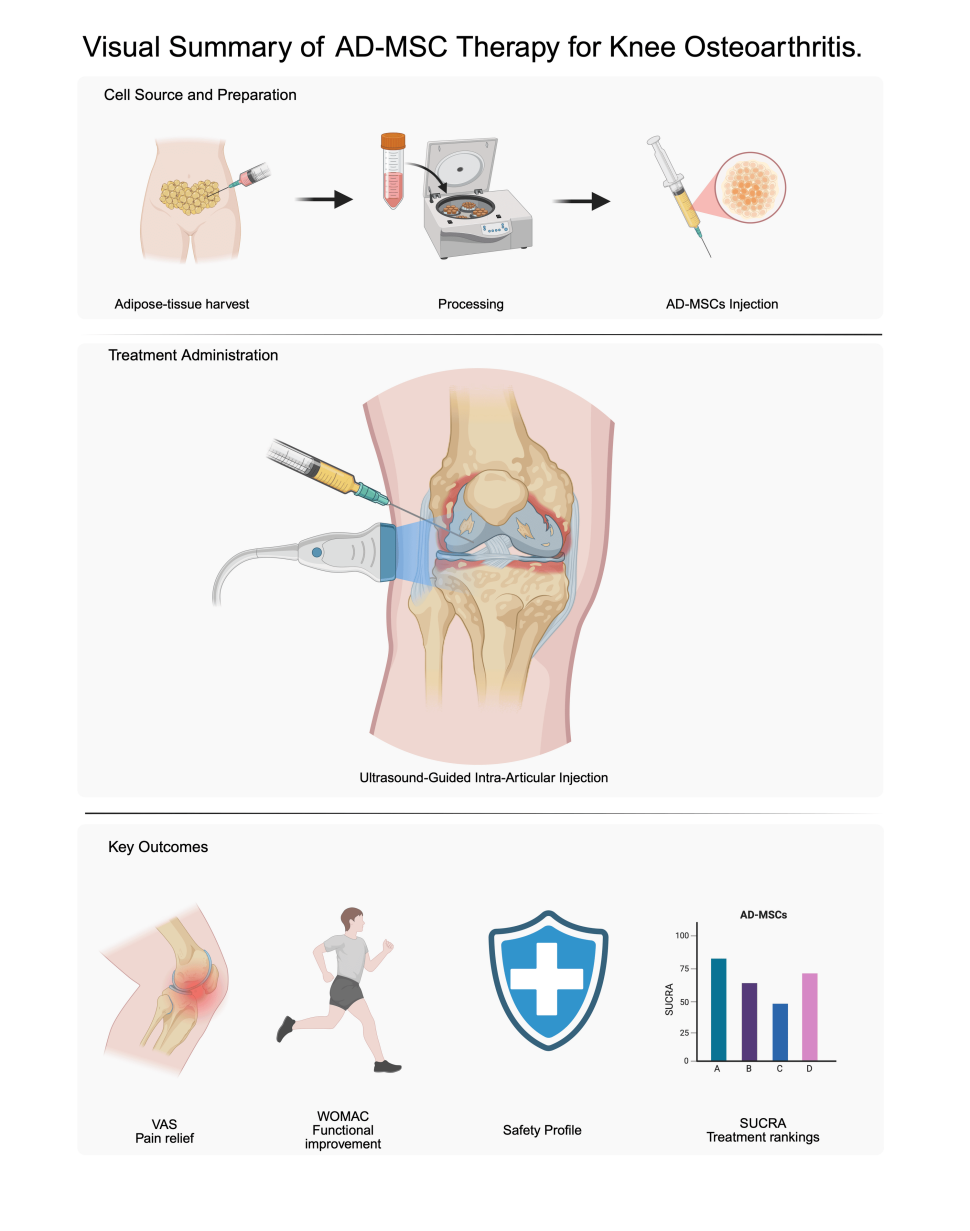
Overview of AD-MSC therapy for knee osteoarthritis AD-MSC: adipose-derived mesenchymal stem cell, VAS: Visual Analog Scale, WOMAC: Western Ontario and McMaster Universities Osteoarthritis Index, SUCRA: Surface Under the Cumulative Ranking Curve Created in BioRender by Alousious Kasagga, https://BioRender.com/i0bfce2

This study aims to generate data-driven insights into the best AD-MSC treatment strategy for knee OA by integrating direct and indirect evidence from multiple RCTs. By doing so, it aims to support the development of standardized protocols for regenerative medicine. Findings from this meta-analysis may also inform future clinical trials and help refine patient selection criteria, ultimately enhancing the therapeutic potential of AD-MSC therapy in OA management. A visual overview of the AD-MSC treatment process and clinical endpoints is presented in Figure 1.

## Review

Methodology

Study Design

This systematic review, traditional pairwise, and network meta-analysis were conducted following the Preferred Reporting Items for Systematic Reviews and Meta-Analyses (PRISMA) 2020 guidelines [9].

The research question was framed using the Population, Intervention, Comparison, and Outcome (PICO) model. The population consisted of adults diagnosed with mild to severe knee osteoarthritis (Kellgren-Lawrence Grade II-IV). The intervention included intra-articular injections of adipose-derived MSCs, categorized into low-dose allogeneic, high-dose allogeneic, and high-dose autologous MSCs. The comparators were placebo and standard care (e.g., corticosteroids, hyaluronic acid injections, physical therapy).

The primary outcomes assessed were pain relief (VAS) and functional improvement (WOMAC) at three, six, and 12 months. Secondary outcomes included treatment durability over time, adverse events, and the necessity for surgical intervention, such as knee replacement.

Inclusion and Exclusion Criteria

This meta-analysis included RCTs evaluating intra-articular AD-MSC therapy for knee OA in adults (≥18 years) diagnosed using validated criteria such as the Kellgren-Lawrence grading system (Grade II-IV). The eligible studies administered adipose-derived MSCs as single or multiple intra-articular injections with defined cell doses and preparation protocols. Comparators included placebo (e.g., saline) and standard care (e.g., hyaluronic acid, corticosteroids, physical therapy). Studies were required to report at least one primary outcome related to pain relief (VAS, Numeric Pain Rating Scale {NPRS}) or functional improvement (WOMAC). Only peer-reviewed RCTs with a minimum six-month follow-up, published in English between January 1, 2010, and January 1, 2025, were included.

Studies were excluded if they involved non-human subjects, pediatric populations, or OA secondary to inflammatory conditions (e.g., rheumatoid arthritis). Trials using MSCs from non-adipose sources or systemic administration were excluded unless adipose-derived MSC outcomes were separately reported. Studies incorporating MSCs with non-standard adjunctive treatments (e.g., gene therapy) or surgical procedures were excluded unless MSC-specific outcomes were independently analyzed. Additionally, non-randomized studies, case reports, reviews, abstracts, preprints, theses, unpublished data, and studies with less than six months of follow-up or published in non-English languages without reliable translations were not considered to maintain methodological rigor.

Search Strategy 

A comprehensive search was conducted across PubMed/MEDLINE, Cochrane Central Register of Controlled Trials (CENTRAL), Embase, ClinicalTrials.gov, Web of Science, and the WHO International Clinical Trials Registry Platform (ICTRP) to identify randomized controlled trials evaluating adipose-derived mesenchymal stem cell therapy for knee osteoarthritis. These databases were selected to ensure broad coverage of published trials across diverse clinical and geographical contexts.

We constructed our search strategy based on two main concepts: AD-MSCs and knee OA. For the AD-MSCs concept, we used keywords such as adipose stem cells, mesenchymal stromal cells, adipose-derived MSCs, adipose tissue stem cells, intra-articular MSC therapy, adipose-derived regenerative cells, stem cell injection, stem cell therapy, and adipose-derived stromal cells. The associated MeSH terms included Mesenchymal Stem Cells [MeSH], Adipose Tissue [MeSH], Stem Cell Transplantation [MeSH], Cell- and Tissue-Based Therapy [MeSH], and Regenerative Medicine [MeSH]. For knee OA, we included keywords such as knee osteoarthritis, osteoarthritis of the knee, degenerative knee joint disease, knee joint arthritis, degenerative joint disease, osteoarthrosis of the knee, and knee cartilage degeneration, and MeSH terms such as Osteoarthritis, Knee [MeSH], Arthritis [MeSH], Knee Joint [MeSH], and Osteoarthritis [MeSH].

These concepts were combined using Boolean operators (AND, OR) to build the advanced search string: (adipose stem cells OR mesenchymal stromal cells OR adipose-derived MSCs OR adipose tissue stem cells OR intra-articular MSC therapy OR adipose-derived regenerative cells OR stem cell injection OR stem cell therapy OR adipose-derived stromal cells OR Mesenchymal Stem Cells [MeSH] OR Adipose Tissue [MeSH] OR Stem Cell Transplantation [MeSH] OR Cell and Tissue-Based Therapy [MeSH] OR Regenerative Medicine [MeSH]) AND (knee osteoarthritis OR osteoarthritis of the knee OR degenerative knee joint disease OR knee joint arthritis OR degenerative joint disease OR osteoarthrosis of the knee OR knee cartilage degeneration OR Osteoarthritis [MeSH] OR Knee [MeSH] OR Arthritis [MeSH] OR Knee Joint [MeSH] OR Osteoarthritis [MeSH]).

The strategy was designed to identify all relevant studies evaluating AD-MSC interventions for knee osteoarthritis up to January 1, 2025, the final search date.

Study Selection

The systematic search across PubMed/MEDLINE, Cochrane CENTRAL, Embase, ClinicalTrials.gov, Web of Science, and WHO ICTRP (World Health Organization International Clinical Trials Registry Platform) identified 994 records. After removing 477 duplicates, 517 records remained for title and abstract screening, of which 461 were excluded for irrelevance. This left 56 full-text reports for eligibility assessment; however, 23 were not retrievable, reducing the total to 33 studies for a full review.

**Figure 2:**
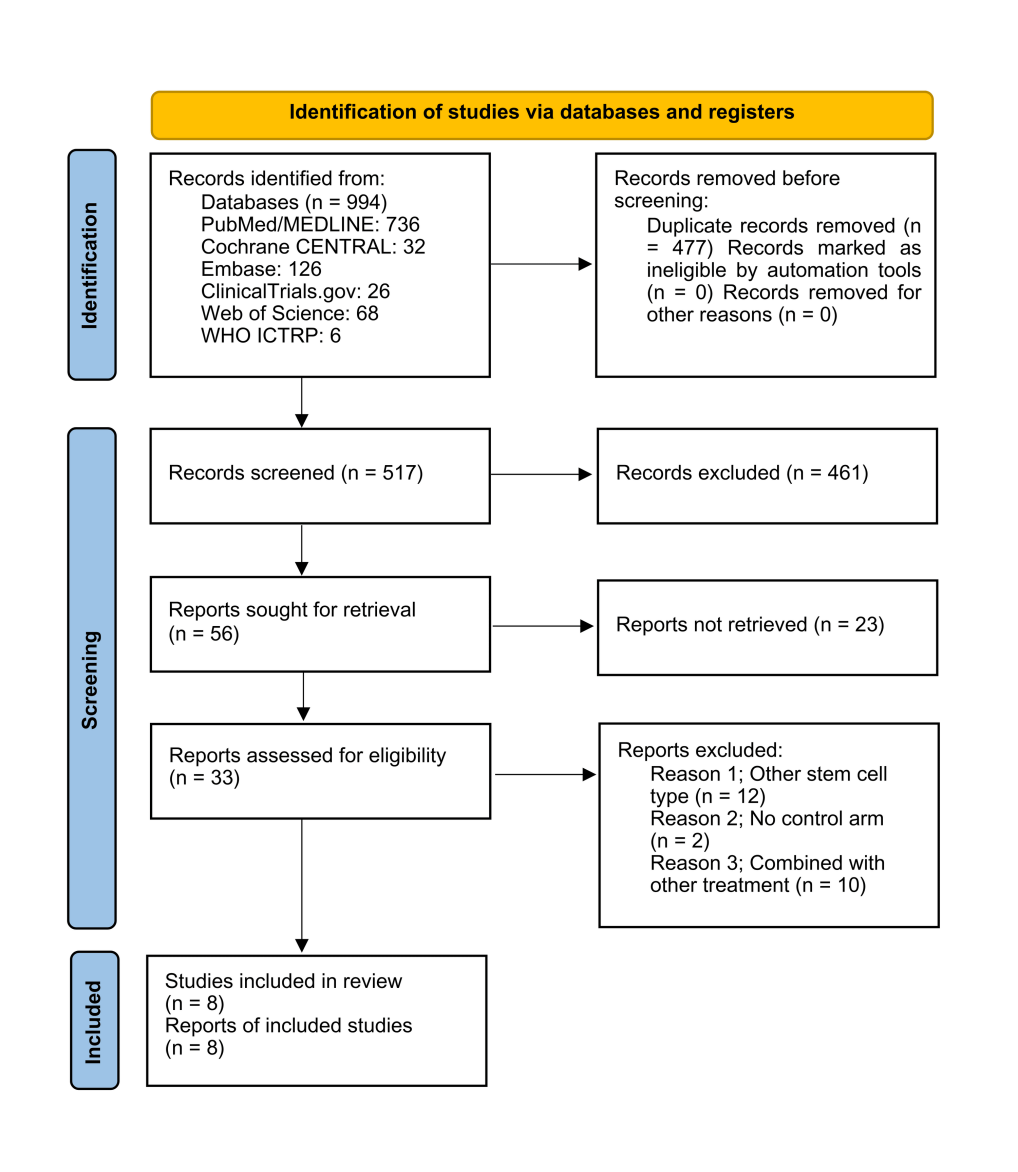
PRISMA flowchart of study selection process PRISMA: Preferred Reporting Items for Systematic Reviews and Meta-Analyses

Following full-text assessment, 25 studies were excluded, primarily due to the use of non-adipose-derived stem cells, the absence of a control arm, or the combination with another intervention. Ultimately, eight RCTs [10-17] met the inclusion criteria and were included in the traditional pairwise and network meta-analyses (Figure 2).

Data Extraction

Data extraction was performed using a Microsoft Excel worksheet (Microsoft Corporation, Redmond, Washington) to ensure consistency and accuracy. Two independent reviewers (Alousious Kasagga and Anushka Verma) gathered data using a predefined form, with any disagreements addressed through discussion or consulting a third reviewer (Eiman Saraya).

Extracted data included study characteristics (authors, year, country, study design), participant demographics (sample size, age, sex, baseline OA severity), and intervention details (MSC dose, frequency, preparation). Comparator treatments (e.g., saline, hyaluronic acid, corticosteroids) were also recorded. 

The primary outcomes included pain relief (VAS, NPRS) and functional improvement (WOMAC), while the secondary outcomes assessed treatment durability, adverse events, and the need for surgical intervention. Follow-up durations and effect estimates (mean differences, risk ratios, 95% confidence intervals) were extracted where available.

Outcome Measurement and Scale Standardization

Pain and function outcomes were assessed using either the VAS or the WOMAC, depending on the reporting format of each included study. For VAS, scores reported on a 0-10 Numeric Pain Rating Scale (NPRS) were converted to a 0-100 scale by multiplying by 10. In cases where the VAS scale was not explicitly specified, assumptions were made based on standard clinical conventions or baseline mean values. For WOMAC, raw scores with a maximum of 96 (sum of pain, stiffness, and function subscales) were converted to a 0-100 scale where applicable to allow for cross-study comparability. If studies reported only subscales, a composite score was derived using standard weighting. All transformations were performed prior to pooling data to minimize measurement-related variability.

Study Characteristics

Eight RCTs were included, evaluating allogeneic and autologous AD-MSCs at varying doses compared to hyaluronic acid, saline, or standard care [10-17]. Table 1 summarizes the primary outcomes, including WOMAC, VAS, Knee injury and Osteoarthritis Outcome Score (KOOS) scores, and adverse events assessed over follow-up periods ranging from one to 24 months.

**Table 1:** Characteristics of included studies (Clinical Outcomes & Measures) WOMAC: Western Ontario and McMaster Universities Osteoarthritis Index, VAS: Visual Analog Scale, KOOS: Knee Injury and Osteoarthritis Outcome Score, SF-36: 36-Item Short Form Survey (Quality of Life Measure), AE: adverse events, SAE: serious adverse events, NPRS: Numeric Pain Rating Scale, MOAKS: MRI Osteoarthritis Knee Score; (Study Design & Interventions) MSC: mesenchymal stem cells, RCT: randomized controlled trial, M/F: male/female distribution, K-L grade: Kellgren-Lawrence grade (osteoarthritis severity classification), Allogeneic: stem cells sourced from a donor, Autologous: stem cells derived from the patient

Author (year)	Study design	MSC dose (#cells)	Mean age (SD)		BMI (Mean ± SD)	Sex distribution		K-L grade (range)	Stem cell source	Comparator	Outcome measures	Follow-up (months)
			Treatment	Control		Treatment: M/F	Control: M/F					
Chen et al. (2021) [10]	RCT	One injection-16M	67.7 (6.84)	70.5 (8.37)	27.65 (3.026)	17 (3/14)	8 (3/5)	II–III	Allogeneic	Hyaluronic acid	WOMAC, VAS, KSCRS, AE, SAE	1, 3, 6, 9, 12, 18, 24
		One injection-32M	68.6 (6.45)		26.72 (4.192)	17 (2/15)						
		One injection-64M	64.9 (4.91)		25.66 (3.782)	15 (3/12)						
		Placebo (hyaluronic)	70.5 (8.37)		25.47 (3.494)	8 (3/5)						
Freitag et al. (2019) [11]	RCT	One injection-100M	54.6 (6.3)	51.5 (6.1)	31.6 (5.9)	10 (4/6)	10 (5/5)	II–III	Autologous	Standard care	NPRS, WOMAC, KOOS, AE, MOAKs	1, 3, 6, 12
		Two injection-100M	54.7 (10.2)		30.4 (5.6)	10 (7/3)						
		Placebo (conservative)	51.5 (6.1)		25.2 (3.4)	10 (5/5)						
Freitag et al. (2024) [12]	RCT	One injection-10M	57.0 (6.7)	39.1 (10.8)	26.30 (3.81)	8 (5/3)	8 (7/1)	II–III	Allogeneic	Plasma-lyte	NRPS, KOOS, AE	1, 3, 6, 9, 12
		One injection-20M	45.5 (12.0)		26.76 (4.66)	8 (6/2)						
		One injection-50M	49.0 (9.6)		26.47 (3.12)	8 (5/3)						
		One injection-100M	47.6 (5.9)		27.59 (4.28)	8 (5/3)						
		Placebo (Plasma-lyte)	39.1 (10.8)		27.81 (3.86)	8 (7/1)						
Kim et al. (2023) [13]	RCT	One injection-100M	63.7 (7.1)	63.8 (7.1)	26.3 (3.2)	125 (39/86)	127 (26/101)	III	Autologous	Saline	WOMAC, VAS, KOOS, SF-36	1, 3, 6
		Placebo (saline)	63.8 (7.1)		25.9 (3.1)	127 (26/101)						
Kuah et al. (2018) [14]	RCT	One injection-3.9M	50.8 (7.3)	55.0 (10.42)	27.7 (2.05)	8 (6/2)	4 (1/3)	I–III	Allogeneic	Cryopreservative	WOMAC, VAS, AE	1, 3, 6, 9, 12
		One injection-6.7M	55.0 (5.15)		26.8 (2.98)	8 (5/3)						
		Placebo	55.0 (10.42)		25.5 (2.84)	4 (1/3)						
Lee et al. (2019) [15]	RCT	One injection-100M	62.2 (6.5)	63.2 (4.2)	25.3 (4.9)	12 (3/9)	12 (3/9)	II–IV	Autologous	saline	WOMAC, VAS, KOOS, SF-36, AE	1, 3, 6
		Placebo (saline)	63.2 (4.2)		25.4 (3.0)	12 (3/9)						
Lu et al. (2019) [16]	RCT	Two injection-50M	55.03 (9.19)	59.64 (5.97)	24.27 (3.04)	26 (3/23)	26 (3/23)	I–III	Autologous	Hyaluronic acid	WOMAC, VAS, SF36	1, 6, 12
		Placebo (hyaluronic)	59.64 (5.97)		24.26 (2.59)	26 (3/23)						
Sadri et al. (2023) [17]	RCT	One injection-100M	52.85 (7.25)	56.1 (7.21)	28.37 (3.26)	20 (2/18)	20 (2/18)	II–III	Allogeneic	Saline	WOMAC, VAS, KOOS, SF-36	1, 3, 6, 12
		Placebo (saline)	56.1 (7.21)		29.12 (4.0)	20 (2/18)						

Table 2 details study methodologies, including randomization, blinding, ethical approval, tissue harvest locations, treatment processes, and funding sources. Most trials utilized double-blinding and were industry-funded, with AD-MSCs primarily harvested from abdominal tissue.

**Table 2:** Study protocols of included trials

Author (year)	Randomization method	Blinding	Ethical approval/trial registration	Tissue harvest location	Treatment process	Funding source
Chen et al. (2021) [10]	Permuted block randomization	Single-blind	Approved, NCT02784964	Not stated	3 ml	Industry and government funded (UnicoCell BioMed Co. Ltd., A+ Industrial Innovative R&D Program, Ministry of Economic Affairs, R.O.C.).
Freitag et al. (2019) [11]	Random number generator	Double-blind	Approved, ACTRN12614000814673	Abdomen	3 ml	Industry and institution funded (Magellan Stem Cells, Melbourne Stem Cell Centre).
Freitag et al. (2024) [12]	Computer-generated blocks	Double-blind	Approved, ACTRN12617001095358	Not stated	5 ml	Industry funded (Magellan Biologicals Pty Ltd.).
Kim et al. (2023) [13]	Permuted block randomization	Double-blind	Approved, NCT03990805	Abdomen	3 ml	Industry funded (R-Bio Co., Ltd.).
Kuah et al. (2018) [14]	Randomization block schedule	Double-blind	Approved, ACTRN12615000439549	Not stated	2 ml	Industry funded (Regeneus Ltd.).
Lee et al. (2019) [15]	Randomization block schedule	Double-blind	Approved, NCT02658344	Abdomen	3 ml	Industry funded (R-Bio Co., Ltd.).
Lu et al. (2019) [16]	computer-generated randomization	Double-blind	Approved, NCT02162693	Abdomen	2.5 ml	Industry and government funded (Cellular Biomedicine Group, National Key Research and Development Program of China).
Sadri et al. (2023) [17]	Permuted block randomization	Triple-blind	Approved, IRCT20080728001031N23	Not stated	5 ml	Government and academic institution funded (Iran Ministry of Health and Medical Education, Royan Institute for Stem Cell Biology and Technology).

Network Structure

The network meta-analysis included eight RCTs with 11 pairwise comparisons across four treatment groups: high-dose autologous AD-MSCs, high-dose allogeneic AD-MSCs, low-dose allogeneic AD-MSCs, and the control group. Some trials featured multiple treatment arms, forming a well-connected network. This network structure forms a cohesive subnetwork, ensuring that indirect comparisons can be made where direct head-to-head trials are unavailable. For instance, since both high-dose autologous and high-dose allogeneic were compared to the control group but not to each other, their relative efficacy can still be estimated through indirect comparison using the standard control group as a reference. By incorporating these comparisons, the analysis provided a comprehensive evaluation of the efficacy and safety of AD-MSC therapies for knee osteoarthritis.

Risk of Bias Assessment

The quality of the included studies was judged using the Cochrane risk of bias 2 (ROB2) tool, incorporating a weighted approach based on each study’s contribution [18]. Two independent reviewers evaluated five domains: randomization, deviations from intended interventions, missing outcome data, outcome measurement, and selection of reported results. Discrepancies were resolved through discussion or consultation with a third reviewer.

Each domain was classified as low risk, with some concerns, or high risk of bias, with overall study ratings determined based on weighted contributions providing a transparent assessment of study quality [19]. Sensitivity analyses were conducted to evaluate the impact of studies with a higher risk of bias on meta-analysis findings.

Statistical Analysis

A traditional pairwise meta-analysis using a fixed-effects model was performed in RevMan 5.4.1 (Review Manager {RevMan}, 2020), with results presented as forest plots for direct treatment comparisons. A frequentist NMA was also performed to estimate overall treatment effects, visualized in a summary frequentist NMA forest plot.

Treatment rankings were determined using a Bayesian NMA framework, generating SUCRA probabilities to evaluate the relative efficacy of interventions [20]. Consistency testing included a frequentist loop inconsistency test and a Bayesian node-splitting analysis to assess direct and indirect evidence agreement. All NMA analyses were performed in MetaInsight (National Institute for Health and Care Research {NIHR} Complex Reviews Support Unit, University of Leicester, UK), following PRISMA-NMA guidelines [21].

Results

Introduction to Results

This section presents findings from the traditional pairwise meta-analysis and network meta-analysis, assessing the effectiveness of different interventions for pain relief and functional improvement, as measured by the VAS and the WOMAC. Analyses were performed at three time points (three, six, and 12 months) to evaluate both short-term and long-term treatment efficacy. Additionally, adverse effects were examined across all time points to assess the safety profile of each intervention.

Traditional meta-analysis (direct comparisons): A traditional pairwise meta-analysis was conducted to summarize direct treatment comparisons from the available studies. The results are presented as forest plots, showing the pooled effect sizes of VAS and WOMAC scores at each time point. Funnel plots were also created to assess publication bias in the direct comparisons.

Frequentist network meta-analysis (indirect and direct comparisons): To provide a comprehensive ranking of treatments, a frequentist NMA was conducted, integrating both direct and indirect evidence. The results are presented using a summary frequentist NMA forest plot, comparing all interventions based on VAS and WOMAC scores at each time point.

Treatment rankings were determined using SUCRA probabilities, derived from a Bayesian framework, which indicates the likelihood of each intervention being the most effective. These rankings are represented through radial plots and Litmus Rank-O-Gram plots, allowing for a clear interpretation of treatment efficacy over time.

Adverse effects analysis: To assess treatment safety, adverse effects were analyzed across all time points. Both traditional meta-analysis and NMA were used to compare adverse effects, with results presented as forest plots displaying risk estimates. Treatments were ranked based on safety profiles using SUCRA values, identifying interventions with the lowest risk of adverse effects.

Validity and quality assessment: To evaluate the reliability of the findings, two additional analyses were conducted. First, a ROB2 assessment was performed to assess study quality and potential bias. The results of the ROB2 evaluation indicate the proportion of studies categorized as having low, some, or high risk of bias, providing insight into the overall credibility of the evidence.

Second, consistency testing was conducted to verify whether direct and indirect evidence agreed within the NMA. A frequentist approach was used to assess global consistency through loop inconsistency tests, while a Bayesian node-splitting analysis was applied to detect potential inconsistencies in specific treatment comparisons.

Summary of treatment rankings: To identify the best-performing treatments, SUCRA values were calculated for VAS and WOMAC scores at all time points. A SUCRA bar graph provides an overview of which treatments are most effective across three, six, and 12 months. These rankings allow for a direct comparison of interventions, facilitating evidence-based decision-making regarding optimal pain management strategies.

**Figure 3:**
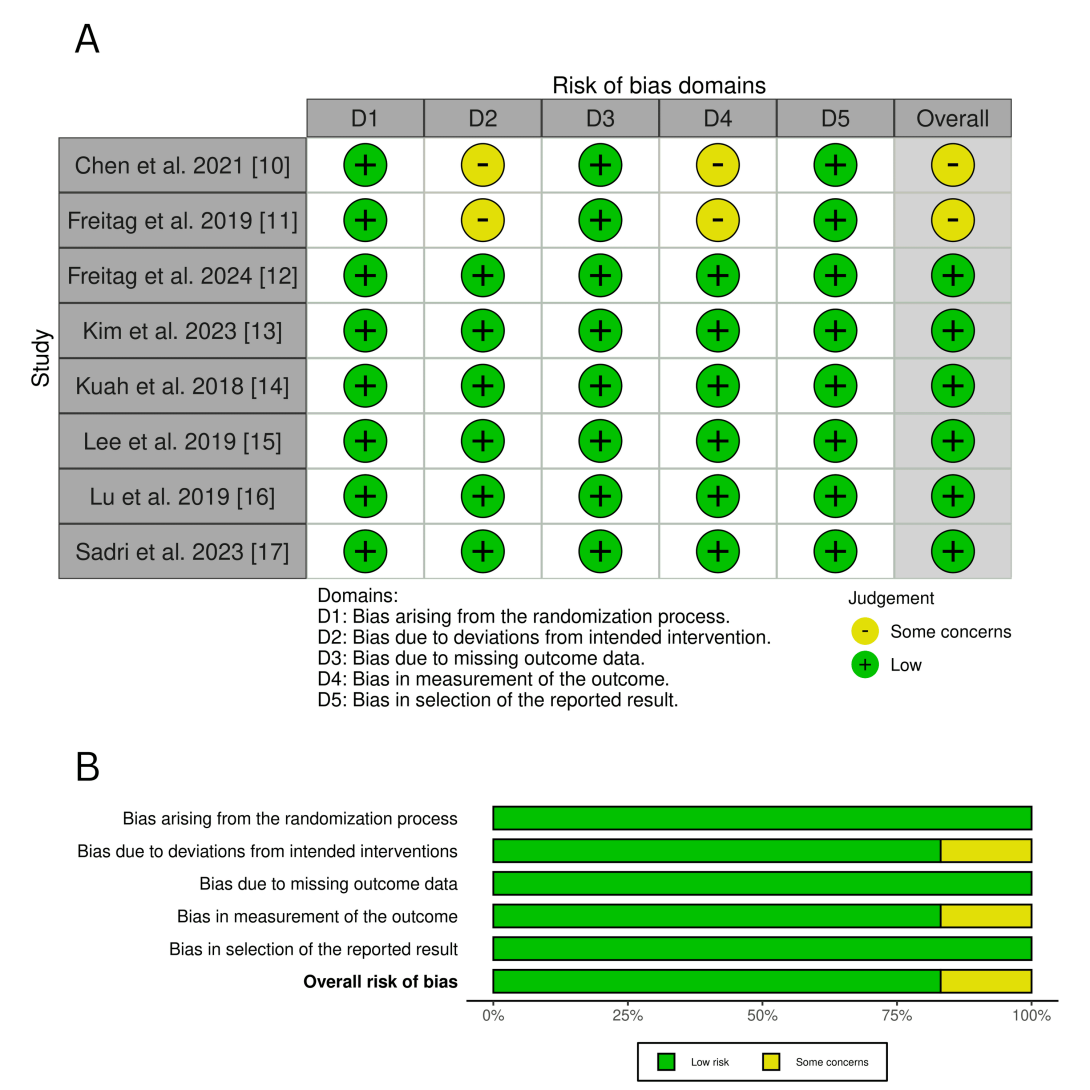
(A) Risk of bias assessment across studies; (B) weighted summary of risk of bias proportions across domains Sources: [10-17]

Risk of bias assessment (ROB2 results): Eight RCTs were assessed using the ROB2 tool, which incorporates a weighted ROB2 approach to account for each study’s contribution to the meta-analysis. As shown in Figure 3A, six studies [12-17] demonstrated a low risk of bias across all domains, while two studies [10,11] showed some concerns in D2 (deviations from intended intervention) and D4 (outcome measurement bias). Figure 3B presents the weighted risk of bias distribution, illustrating that most studies had a low risk of bias. Some concerns were noted in 25% of studies for D2 and D4, but their impact was minimal.

VAS Score Change (Three Months)

Traditional meta-analysis: A traditional pairwise meta-analysis using a fixed-effects model evaluated the change in VAS scores at three months across three treatment groups: low-dose allogeneic, high-dose allogeneic, and high-dose autologous AD-MSCs. A total of 270 participants were analyzed across eight studies.

**Figure 4:**
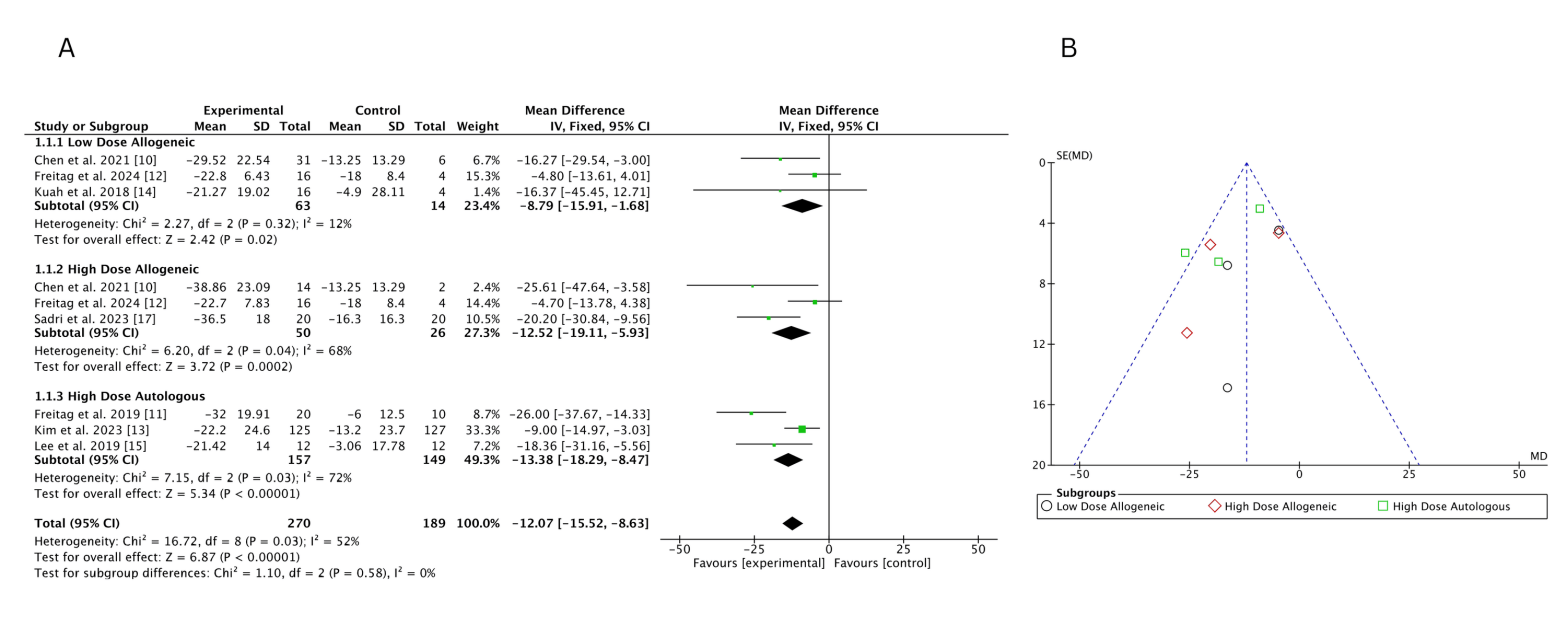
(A) Forest plot of direct comparisons for VAS score change at three months; (B) funnel plot assessing publication bias VAS: Visual Analog Scale Sources: [10-15,17]

High-dose autologous AD-MSCs demonstrated the greatest improvement in VAS scores (MD = -13.38; 95% CI: -18.29, -8.47), followed by high-dose allogeneic AD-MSCs (MD = -12.52; 95% CI: -19.11, -5.93). Low-dose allogeneic AD-MSCs showed the smallest effect (MD = -8.79; 95% CI: -15.91, -1.68). Heterogeneity was low for low-dose allogeneic (I² = 12%) but higher for high-dose allogeneic (I² = 68%) and high-dose autologous (I² = 72%), indicating some variability in treatment effects, as shown in Figure 4A. The funnel plot, Figure 4B, suggests no strong evidence of publication bias, as most studies are symmetrically distributed. However, a slight asymmetry is observed for high-dose allogeneic AD-MSCs, suggesting potential small-study effects.

Frequentist network meta-analysis: A frequentist NMA was conducted to compare the effectiveness of low-dose allogeneic, high-dose allogeneic, and high-dose autologous AD-MSCs in reducing VAS scores at three months. The estimated mean differences (MD) with 95% confidence intervals (CI) for both direct and indirect comparisons are summarized below.

Low-dose allogeneic AD-MSCs showed the greatest improvement in VAS scores in Chen et al. (2021) (MD = -16.27; 95% CI: -28.42, -4.12), while Freitag et al. (2024) and Kuah et al. (2018) reported smaller effects with wide confidence intervals, indicating uncertainty in their estimates [10,12,14]. High-dose allogeneic AD-MSCs had the most substantial pain reduction in Chen et al. (2021) (MD = -25.61; 95% CI: -40.81, -10.41), whereas Freitag et al. (2024) and Sadri et al. (2023) showed more variable responses, with some confidence intervals crossing zero [10,12,17]. When comparing high-dose allogeneic to low-dose allogeneic AD-MSCs, the effect size was less pronounced (MD = -9.34; 95% CI: -23.81, 5.13), suggesting comparable efficacy between the two treatments. High-dose autologous AD-MSCs, particularly in Freitag et al. (2019), exhibited the strongest VAS reduction (MD = -26.00; 95% CI: -37.67, -14.33), with similar but slightly less pronounced effects observed in Kim et al. (2023) and Lee et al. (2019) [11,13,15]. 

**Figure 5:**
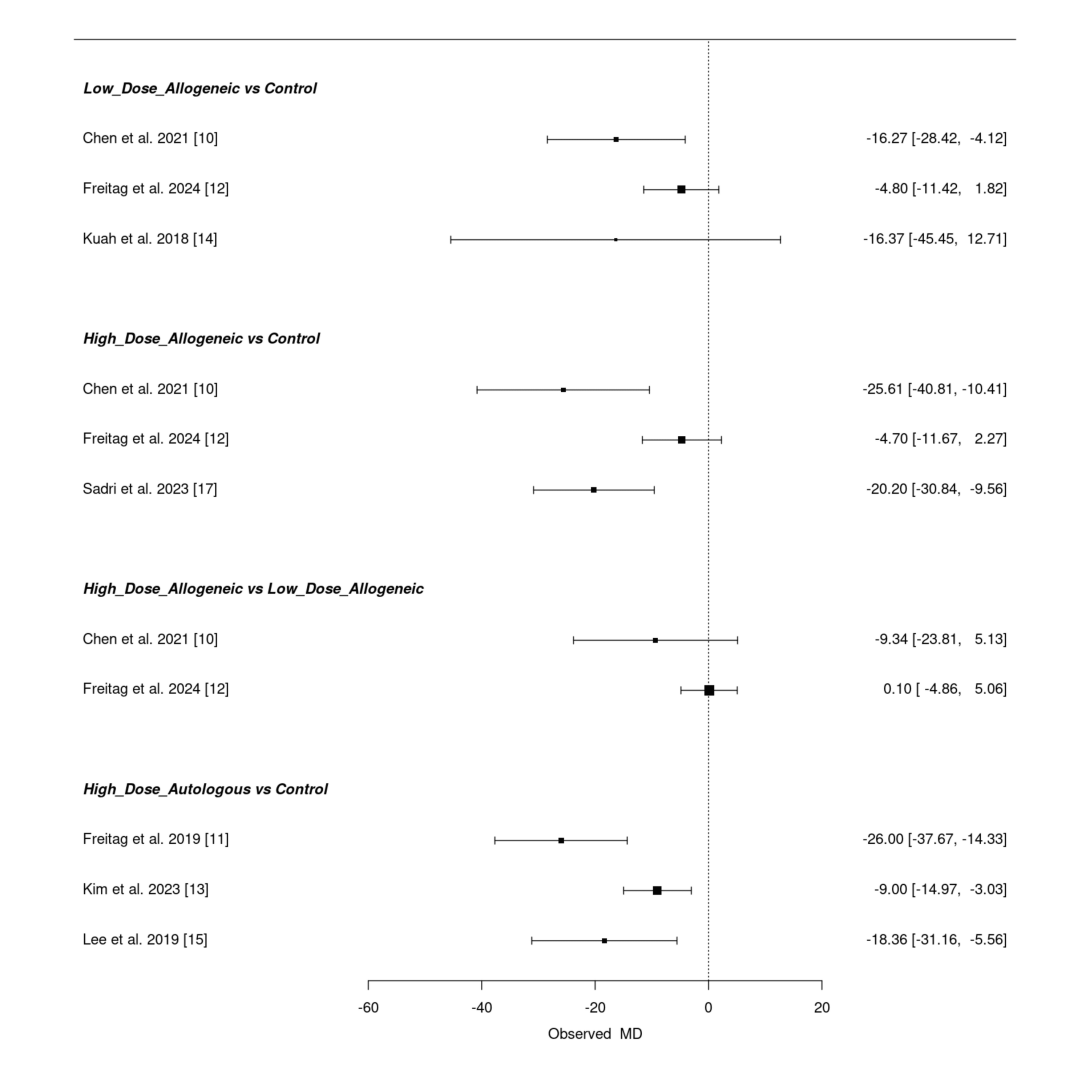
Summary frequentist NMA forest plot for VAS score change at three months NMA: network meta-analysis, VAS: Visual Analog Scale Sources: [10-15,17]

Overall, high-dose autologous AD-MSCs consistently showed the largest VAS reduction, followed by high-dose allogeneic AD-MSCs. However, some comparisons exhibited wide confidence intervals, indicating uncertainty in certain pooled estimates. Figure 5 presents the summary NMA forest plot, visually displaying the relative treatment effects and their confidence intervals.

SUCRA rankings for VAS score at three months: A SUCRA analysis was performed to rank the effectiveness of low-dose allogeneic, high-dose allogeneic, and high-dose autologous AD-MSCs in reducing VAS scores at three months. 

**Figure 6:**
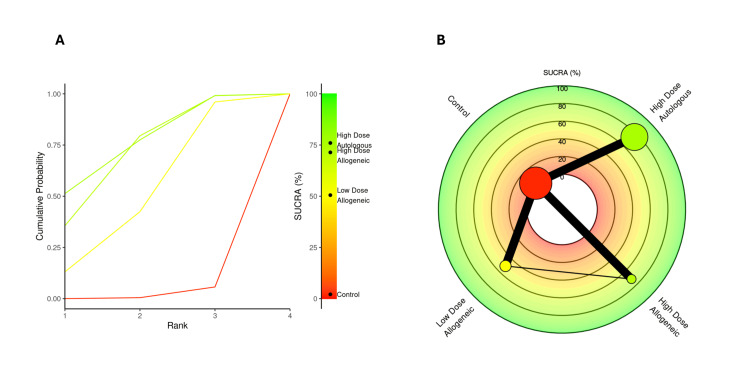
SUCRA rank plots for VAS score at three months: (A) radial rank plot – displays cumulative probability ranking for each treatment; (B) Litmus Rank-O-Gram – highlights the relative probability of each treatment being the most effective SUCRA: Surface Under the Cumulative Ranking, VAS: Visual Analog Scale

High-dose autologous AD-MSCs ranked highest (SUCRA: 75.99%), followed by high-dose allogeneic AD-MSCs (SUCRA: 71.39%), indicating strong pain reduction effects for both treatments. Low-dose allogeneic AD-MSCs ranked moderately (SUCRA: 50.53%), while the control group had the lowest SUCRA score (2.08%), reinforcing its inferiority compared to AD-MSCs interventions. The radial rank plot, Figure 6A, illustrates the cumulative probability of each treatment being ranked best, with high-dose autologous AD-MSCs displaying the steepest curve, indicating a higher likelihood of being the most effective treatment. The Litmus Rank-O-Gram, Figure 6B, further confirms that high-dose autologous AD-MSCs occupy the highest probability region while the control group remains in the lowest ranking zone.

WOMAC Score Change (Three Months)

Traditional meta-analysis: At three months, a fixed-effects pairwise meta-analysis was conducted to evaluate WOMAC score changes across low-dose allogeneic, high-dose allogeneic, and high-dose autologous AD-MSCs. A total of 270 participants were included.

**Figure 7:**
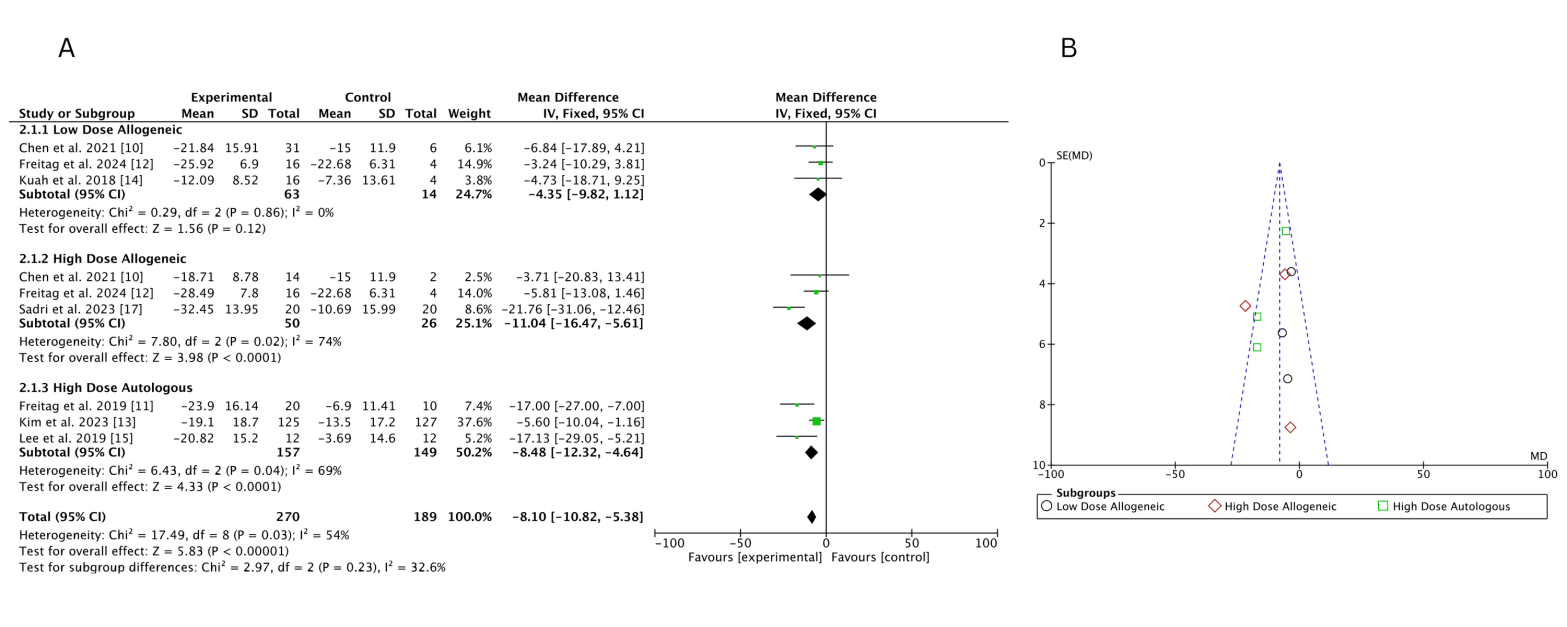
(A) Forest plot of direct comparisons for WOMAC score change at three months; (B) funnel plot assessing publication bias WOMAC: Western Ontario and McMaster Universities Osteoarthritis Index Sources: [10-15,17]

High-dose allogeneic AD-MSCs showed the largest improvement (MD = -11.04; 95% CI: -16.47, -5.61), followed by high-dose autologous AD-MSCs (MD = -8.48; 95% CI: -12.32, -4.64). Low-dose allogeneic AD-MSCs had the smallest effect (MD = -4.35; 95% CI: -9.82, 1.12) and did not show a statistically significant difference compared to the control (p = 0.12). Heterogeneity was low for low-dose allogeneic AD-MSCs (I² = 0%) but moderate to high for high-dose allogeneic (I² = 74%) and high-dose autologous (I² = 69%), indicating some variability among studies, as shown in Figure 7A. The funnel plot, Figure 7B, showed a relatively symmetrical distribution, with some asymmetry for high-dose allogeneic AD-MSCs, suggesting potential small-study effects but no major publication bias.

Frequentist network meta-analysis: A frequentist NMA was conducted to compare the effectiveness of low-dose allogeneic, high-dose allogeneic, and high-dose autologous AD-MSCs in reducing WOMAC scores at three months. The estimated mean differences (MD) with 95% confidence intervals (CI) for both direct and indirect comparisons are summarized below.

Low-dose allogeneic AD-MSCs showed modest effects, with Chen et al. (2021) (MD = -6.46; 95% CI: -16.43, 3.51) and Freitag et al. (2024) (MD = -3.24; 95% CI: -8.77, 2.29) reporting small improvements [10,12]. Kuah et al. (2018) (MD = -4.73; 95% CI: -18.70, 9.25) showed wide confidence intervals, reflecting variability in effect estimates [14]. High-dose allogeneic AD-MSCs demonstrated greater reductions in WOMAC scores, with Sadri et al. (2023) (MD = -21.76; 95% CI: -31.06, -12.46) showing the most substantial improvement [17]. Freitag et al. (2024) (MD = -5.81; 95% CI: -11.62, -0.00) showed a smaller but still significant reduction, while Chen et al. (2021) (MD = -3.33; 95% CI: -12.78, 6.12) had a wide confidence interval, indicating uncertainty [10,12].

When comparing high-dose allogeneic to low-dose allogeneic AD-MSCs, the results were inconclusive, with Chen et al. (2021) (MD = 3.13; 95% CI: -4.12, 10.38) and Freitag et al. (2024) (MD = -2.57; 95% CI: -7.67, 2.53) showing overlapping confidence intervals [10,12]. High-dose autologous MSCs showed the strongest WOMAC score reduction, with Freitag et al. (2019) (MD = -17.00; 95% CI: -27.00, -7.00) and Lee et al. (2019) (MD = -17.13; 95% CI: -29.05, -5.21) demonstrating significant improvements, while Kim et al. (2023) (MD = -5.60; 95% CI: -10.04, -1.16) showed a moderate effect [11,13,15]. 

**Figure 8:**
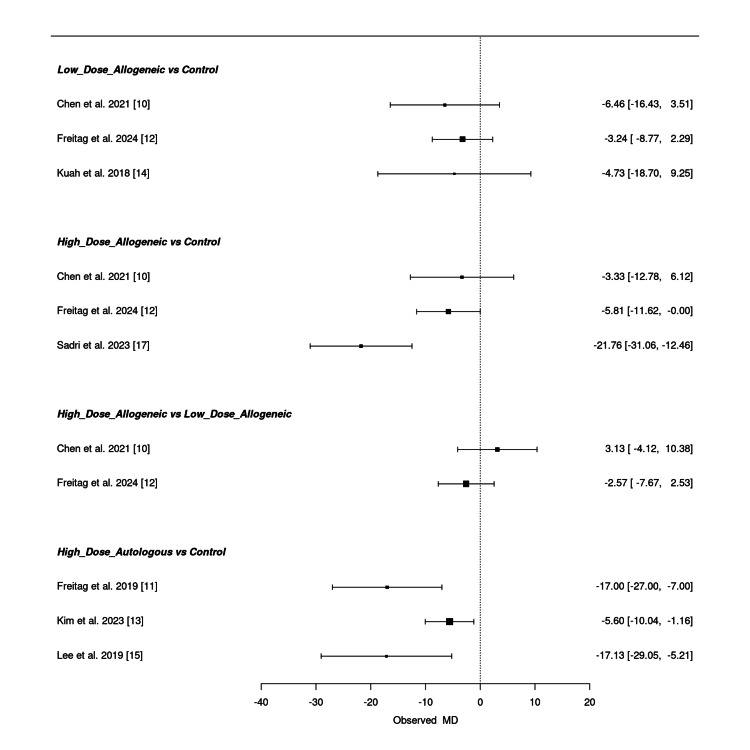
Summary frequentist NMA forest plot for WOMAC score change at three months NMA: network meta-analysis, WOMAC: Western Ontario and McMaster Universities Osteoarthritis Index Sources: [10-15,17]

Overall, high-dose autologous AD-MSCs consistently provided the largest WOMAC score reductions, followed by high-dose allogeneic AD-MSCs, while low-dose allogeneic AD-MSCs showed inconsistent effects. Some comparisons had wide confidence intervals, suggesting uncertainty in certain pooled estimates. Figure 8 presents the summary NMA forest plot, visually displaying the relative treatment effects with their corresponding confidence intervals.

SUCRA rankings for WOMAC score at three months: A SUCRA analysis was conducted to evaluate the effectiveness of low-dose allogeneic, high-dose allogeneic, and high-dose autologous AD-MSCs in improving WOMAC scores at three months. 

**Figure 9:**
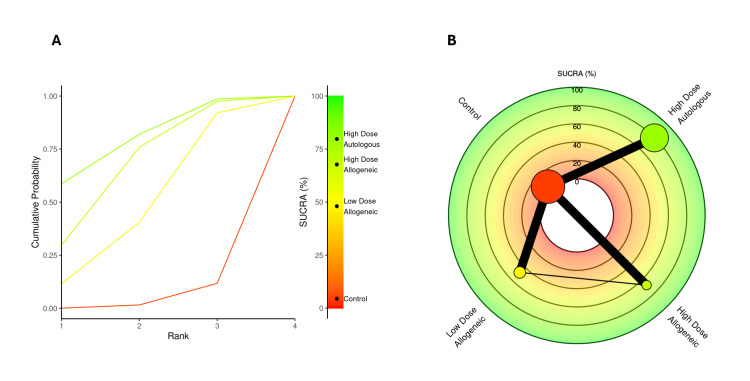
SUCRA rank plots for WOMAC score at three months: (A) radial rank plot – displays cumulative probability ranking for each treatment; (B) Litmus Rank-O-Gram – highlights the relative probability of each treatment being the most effective SUCRA: Surface Under the Cumulative Ranking, WOMAC: Western Ontario and McMaster Universities Osteoarthritis Index

High-dose autologous AD-MSCs ranked highest (SUCRA: 79.75%), followed by high-dose allogeneic AD-MSCs (SUCRA: 67.72%), confirming their strong impact on WOMAC score improvements. Low-dose allogeneic AD-MSCs ranked lower (SUCRA: 48.06%), while the control group had the lowest SUCRA score (4.46%), reinforcing its limited effectiveness in reducing symptoms. The radial rank plot, Figure 9A, illustrates the cumulative probability of each treatment being the best-ranked intervention, with high-dose autologous AD-MSCs showing the steepest curve, confirming its superior effectiveness. The Litmus Rank-O-Gram, Figure 9B, further highlights the probability distribution, with high-dose autologous AD-MSCs consistently ranked the highest.

VAS Score Change (Six Months)

Traditional meta-analysis: At six months, VAS score changes were analyzed using fixed-effects pairwise meta-analysis, comparing low-dose allogeneic, high-dose allogeneic, and high-dose autologous AD-MSCs. A total of 300 participants from multiple studies were analyzed.

**Figure 10:**
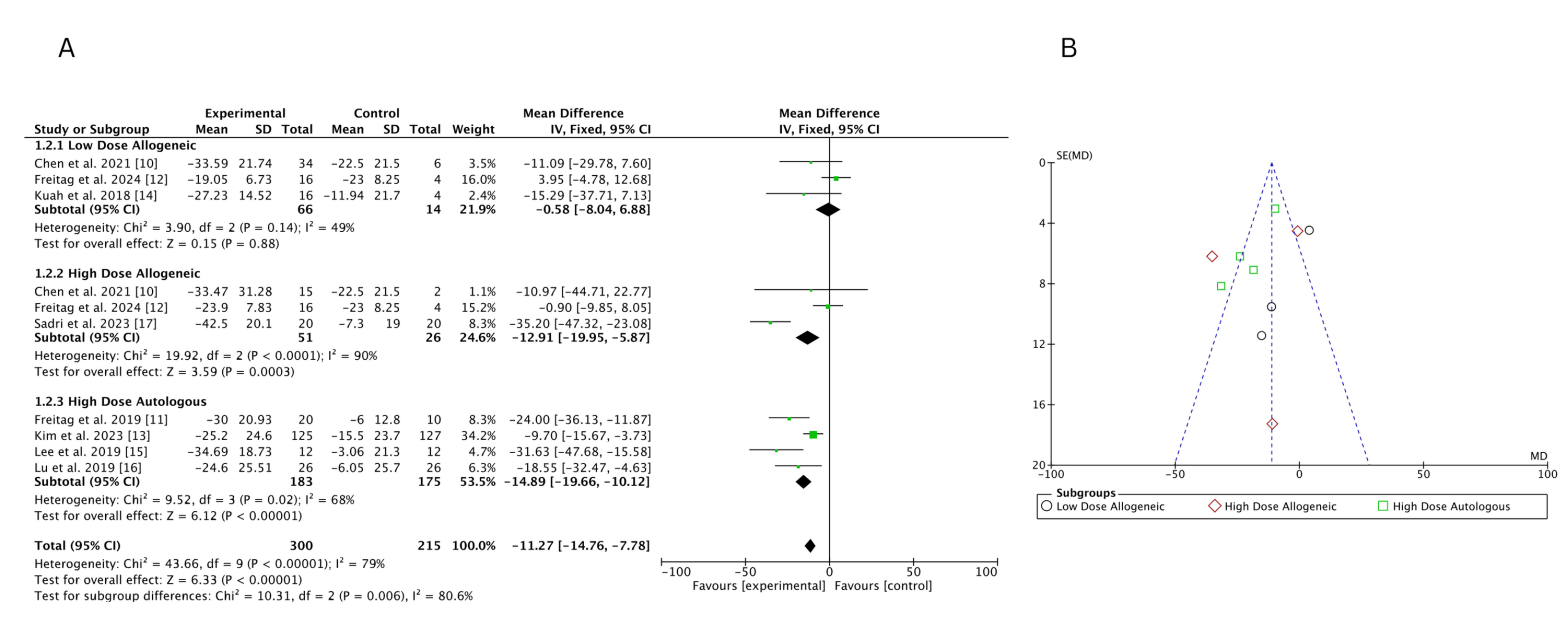
(A) Forest plot of direct comparisons for VAS score change at six months; (B) funnel plot assessing publication bias VAS: Visual Analog Scale Sources: [10-17]

High-dose autologous AD-MSCs continued to show the greatest improvement (MD = -14.89; 95% CI: -19.66, -10.12), followed by high-dose allogeneic MSCs (MD = -12.91; 95% CI: -19.95, -5.87). Low-dose allogeneic AD-MSCs showed minimal effect (MD = -0.58; 95% CI: -8.04, 6.88), with results overlapping the null effect line, indicating no significant benefit over control. Heterogeneity remained high for high-dose allogeneic (I² = 90%) and high-dose autologous (I² = 68%), while low-dose allogeneic (I² = 49%) showed moderate heterogeneity, as shown in Figure 10A. The funnel plot, Figure 10B, suggests potential asymmetry, particularly for high-dose allogeneic AD-MSCs, indicating possible small-study effects. However, overall distribution remains mostly balanced, reducing concerns of major publication bias.

Frequentist network meta-analysis: A frequentist NMA was conducted to evaluate the effectiveness of low-dose allogeneic, high-dose allogeneic, and high-dose autologous AD-MSCs in reducing VAS scores at six months. The estimated mean differences (MD) with 95% confidence intervals (CI) for both direct and indirect comparisons are summarized below.

Low-dose allogeneic AD-MSCs showed varying effects across studies. Chen et al. (2021) (MD = -11.09; 95% CI: -27.68, 5.50) reported a moderate reduction, whereas Freitag et al. (2024) (MD = 3.95; 95% CI: -2.65, 10.55) showed a slight positive estimate, and Kuah et al. (2018) (MD = -15.29; 95% CI: -37.71, 7.13) had a wide confidence interval, indicating uncertainty in the pooled effect [10,12,14]. High-dose allogeneic AD-MSCs demonstrated a substantial reduction in VAS scores in Sadri et al. (2023) (MD = -35.20; 95% CI: -47.38, -23.02), while Chen et al. (2021) (MD = -10.97; 95% CI: -32.71, 10.77) and Freitag et al. (2024) (MD = -0.90; 95% CI: -7.78, 5.98) had less consistent effects [10,12,17]. The comparison between high-dose allogeneic and low-dose allogeneic AD-MSCs was relatively small, with Chen et al. (2021) (MD = 0.12; 95% CI: -17.32, 17.56) and Freitag et al. (2024) (MD = -4.85; 95% CI: -9.91, 0.21) showing overlapping confidence intervals [10,12]. High-dose autologous AD-MSCs exhibited the strongest reduction in VAS scores. Freitag et al. (2019) (MD = -24.00; 95% CI: -36.13, -11.87) and Lee et al. (2019) (MD = -31.63; 95% CI: -47.68, -15.58) showed substantial effects, while Kim et al. (2023) (MD = -9.70; 95% CI: -15.67, -3.73) and Lu et al. (2019) (MD = -18.55; 95% CI: -32.37, -4.73) reported more moderate reductions [11,13,15,16].

**Figure 11:**
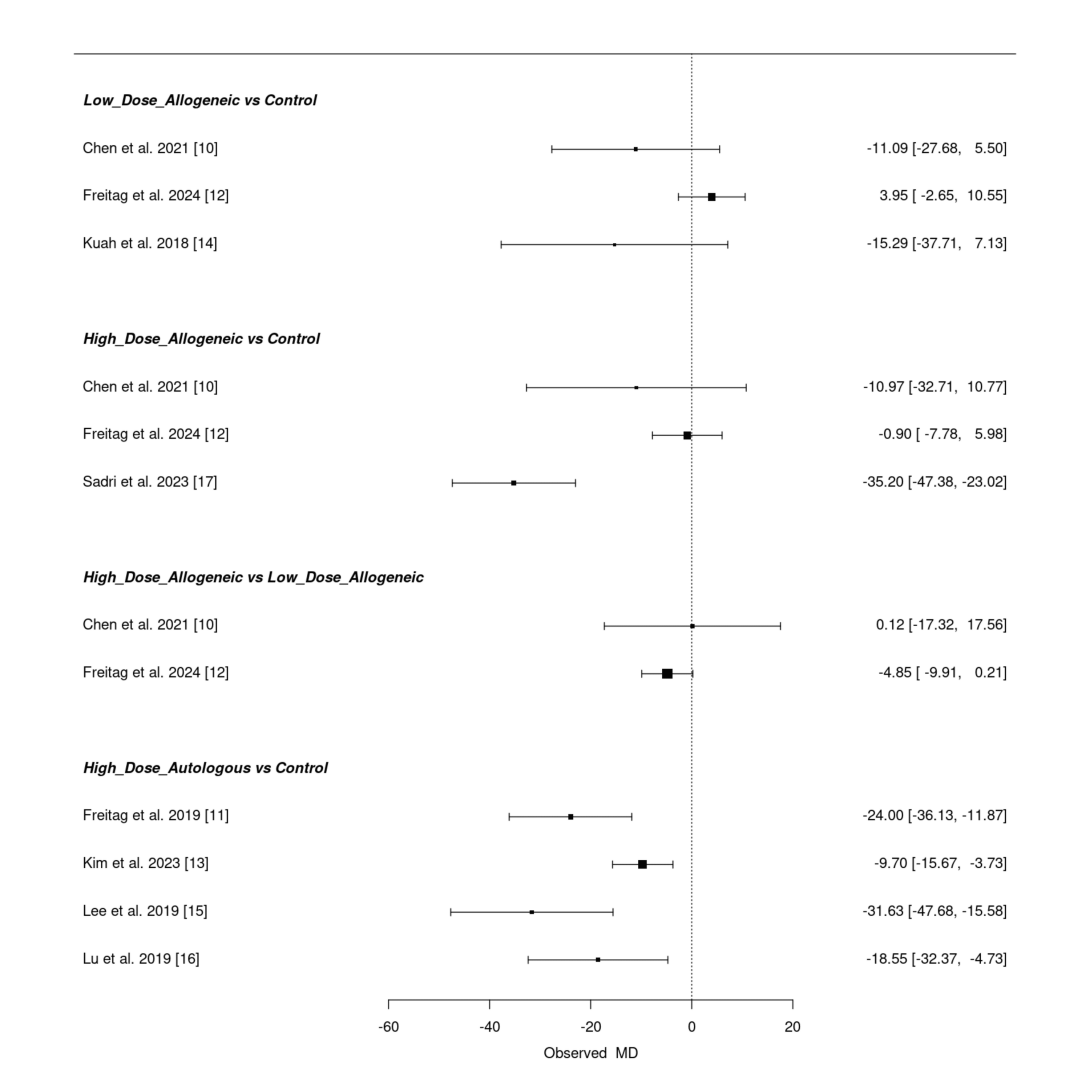
Summary frequentist NMA forest plot for VAS score change at six months NMA: network meta-analysis, VAS: Visual Analog Scale Sources: [10-17]

Overall, high-dose autologous AD-MSCs consistently showed the most significant pain reduction, followed by high-dose allogeneic AD-MSCs, though some comparisons had wide confidence intervals, suggesting uncertainty in certain estimates. Figure 11 presents the summary NMA forest plot, visually displaying the relative treatment effects with their corresponding confidence intervals.

SUCRA rankings for VAS score at six months: A SUCRA analysis was conducted to rank the effectiveness of low-dose allogeneic, high-dose allogeneic, and high-dose autologous AD-MSCs in reducing VAS scores at six months. 

**Figure 12:**
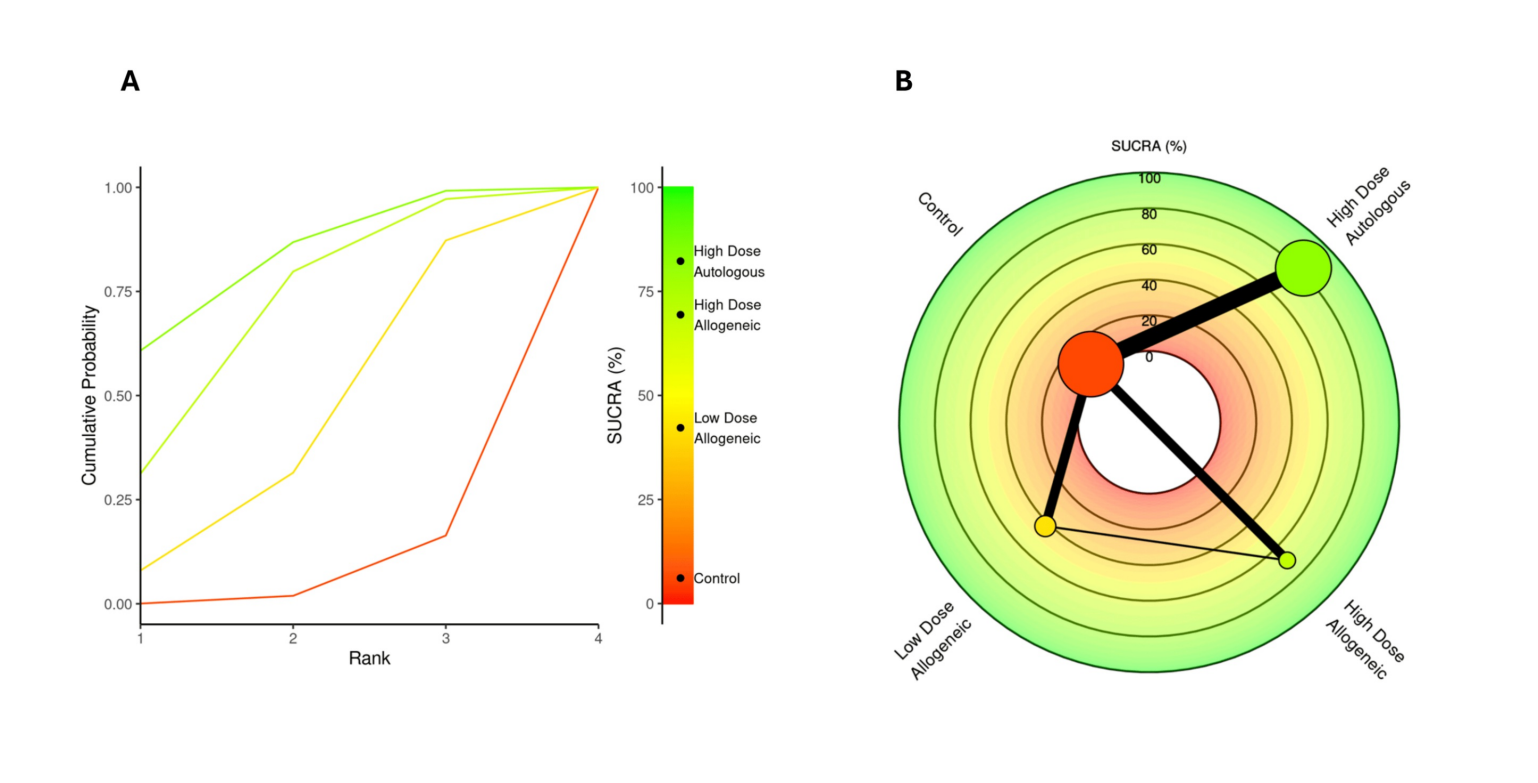
SUCRA rank plots for VAS score at six months: (A) radial rank plot – displays cumulative probability ranking for each treatment; (B) Litmus Rank-O-Gram – highlights the relative probability of each treatment being the most effective SUCRA: Surface Under the Cumulative Ranking, VAS: Visual Analog Scale

High-dose autologous AD-MSCs ranked highest (SUCRA: 82.27%), maintaining its position as the most effective treatment. High-dose allogeneic AD-MSCs followed (SUCRA: 69.39%), while low-dose allogeneic AD-MSCs ranked lower (SUCRA: 42.23%). The control group had the lowest SUCRA score (6.11%), reinforcing its inferiority. The radial rank plot, Figure 12A, illustrates the cumulative probability of each treatment being ranked best, showing a clear separation of high-dose autologous AD-MSCs as the most effective option. The Litmus Rank-O-Gram, Figure 12B, further supports this finding, highlighting the relative ranking probability of each treatment.

WOMAC Score Change (Six Months)

Traditional meta-analysis: At six months, a fixed-effects pairwise meta-analysis evaluated WOMAC score changes across low-dose allogeneic, high-dose allogeneic, and high-dose autologous AD-MSCs, including 300 participants.

**Figure 13:**
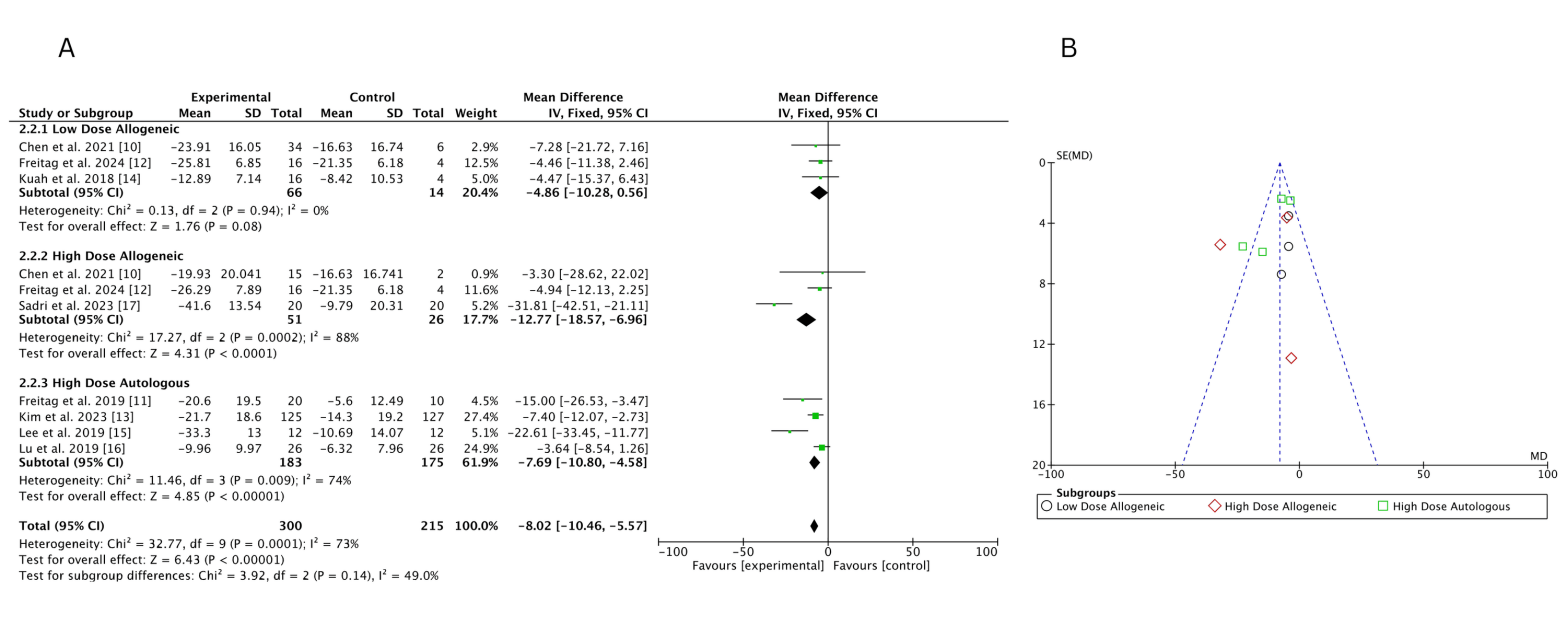
(A) Forest plot of direct comparisons for WOMAC score change at six months; (B) funnel plot assessing publication bias WOMAC: Western Ontario and McMaster Universities Osteoarthritis Index Sources: [10-17]

High-dose allogeneic AD-MSCs showed the greatest improvement in WOMAC scores at six months (MD = -12.77; 95% CI: -18.57, -6.96), followed by high-dose autologous AD-MSCs (MD = -7.69; 95% CI: -10.80, -4.58). Low-dose allogeneic MSCs showed no significant improvement compared to control (MD = -4.86; 95% CI: -10.28, 0.56; p = 0.08). Heterogeneity was high for high-dose allogeneic MSCs (I² = 88%) and moderate for high-dose autologous AD-MSCs (I² = 74%), while low-dose allogeneic AD-MSCs (I² = 0%) showed no heterogeneity, as illustrated in Figure 13A. The funnel plot, Figure 13B, suggests slight asymmetry in high-dose allogeneic AD-MSCs, indicating potential small-study effects but no major publication bias.

Frequentist network meta-analysis: A frequentist NMA was conducted to evaluate the effectiveness of low-dose allogeneic, high-dose allogeneic, and high-dose autologous AD-MSCs in reducing WOMAC scores at six months. The estimated mean differences (MD) with 95% confidence intervals (CI) for both direct and indirect comparisons are summarized below.

Low-dose allogeneic AD-MSCs showed inconsistent effects across studies. Freitag et al. (2024) (MD = -4.46; 95% CI: -9.90, 0.98) and Kuah et al. (2018) (MD = -4.47; 95% CI: -15.37, 6.43) exhibited wide confidence intervals, suggesting variability in treatment response [12,14]. Chen et al. (2021) (MD = -7.28; 95% CI: -20.07, 5.51) also showed a high degree of uncertainty, indicating that low-dose allogeneic AD-MSCs may not provide a significant improvement over control [10]. High-dose allogeneic AD-MSCs demonstrated stronger WOMAC score reductions, particularly in Sadri et al. (2023) (MD = -31.81; 95% CI: -42.51, -21.11) [17]. Freitag et al. (2024) (MD = -4.94; 95% CI: -10.71, 0.83) and Chen et al. (2021) (MD = -3.30; 95% CI: -18.71, 12.11) showed smaller effects with overlapping confidence intervals, suggesting uncertainty in the pooled results [10,12]. When comparing high-dose allogeneic to low-dose allogeneic AD-MSCs, the effect estimates were inconclusive, with Chen et al. (2021) (MD = 3.98; 95% CI: -7.51, 15.47) showing a non-significant positive estimate and Freitag et al. (2024) (MD = -0.48; 95% CI: -5.60, 4.64) showing no clear difference between treatments [10,12]. High-dose autologous MSCs showed the strongest WOMAC score reductions, with Lee et al. (2019) (MD = -22.61; 95% CI: -33.45, -11.77) and Freitag et al. (2019) (MD = -15.00; 95% CI: -26.53, -3.47) reporting significant pain improvements [11,15]. Kim et al. (2023) (MD = -7.40; 95% CI: -12.07, -2.73) showed a more moderate reduction, while Lu et al. (2019) (MD = -3.64; 95% CI: -8.54, 1.26) had a confidence interval crossing zero, indicating potential treatment variability [13,16].

**Figure 14:**
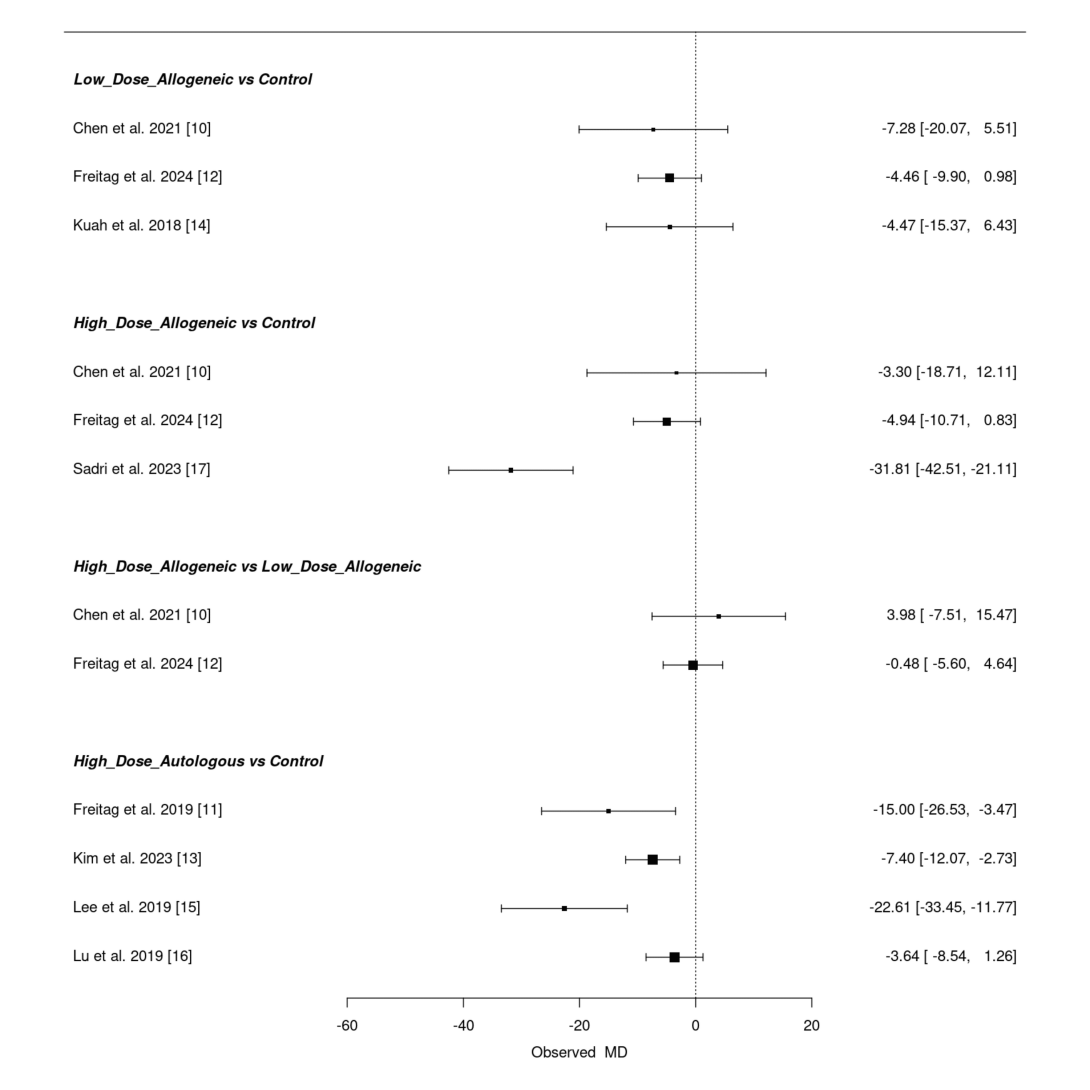
Summary frequentist NMA forest plot for WOMAC score change at six months NMA: network meta-analysis, WOMAC, Western Ontario and McMaster Universities Osteoarthritis Index Sources: [10-17]

Overall, high-dose autologous AD-MSCs provided the most consistent WOMAC score reductions, followed by high-dose allogeneic AD-MSCs, while low-dose allogeneic AD-MSCs showed uncertain effects. Some comparisons had wide confidence intervals, reflecting variability in the pooled estimates. Figure 14 presents the summary NMA forest plot, visually displaying the relative treatment effects and their corresponding confidence intervals.

SUCRA rankings for WOMAC score at six months: A SUCRA analysis was performed to rank the effectiveness of low-dose allogeneic, high-dose allogeneic, and high-dose autologous AD-MSCs in improving WOMAC scores at six months. 

**Figure 15:**
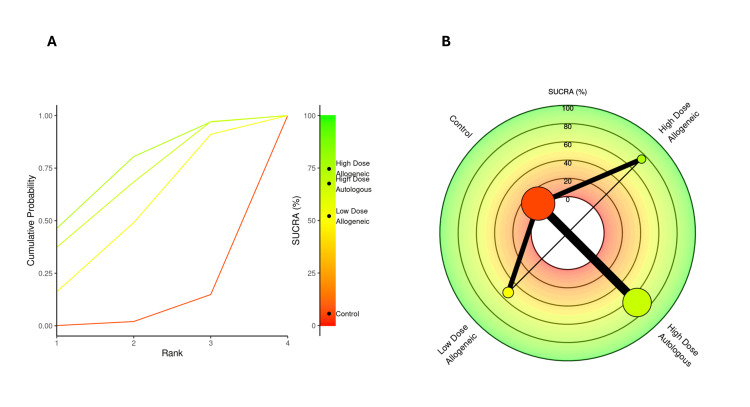
SUCRA rank plots for WOMAC score at six months: (A) radial rank plot – displays cumulative probability ranking for each treatment; (B) Litmus Rank-O-Gram – highlights the relative probability of each treatment being the most effective SUCRA: Surface Under the Cumulative Ranking Curve, WOMAC: Western Ontario and McMaster Universities Osteoarthritis Index

High-dose allogeneic AD-MSCs ranked highest (SUCRA: 74.59%), surpassing high-dose autologous AD-MSCs (SUCRA: 67.62%), which had previously ranked higher at three months. Low-dose allogeneic AD-MSCs ranked moderately (SUCRA: 52.11%), while the control group had the lowest SUCRA score (5.67%), reinforcing its continued lack of efficacy. The radial rank plot, Figure 15A, illustrates the cumulative probability of each treatment being the best-ranked intervention, with high-dose allogeneic AD-MSCs demonstrating the steepest curve, indicating improved effectiveness over time. The Litmus Rank-O-Gram, Figure 15B, further confirms the ranking probability distribution, showing the dominance of both high-dose MSC interventions.

VAS Score Change (12 Months)

Traditional meta-analysis:* *At 12 months, VAS score changes were analyzed using a fixed-effects pairwise meta-analysis, comparing the effects of low-dose allogeneic, high-dose allogeneic, and high-dose autologous AD-MSCs. A total of 163 participants were included across multiple studies.

**Figure 16:**
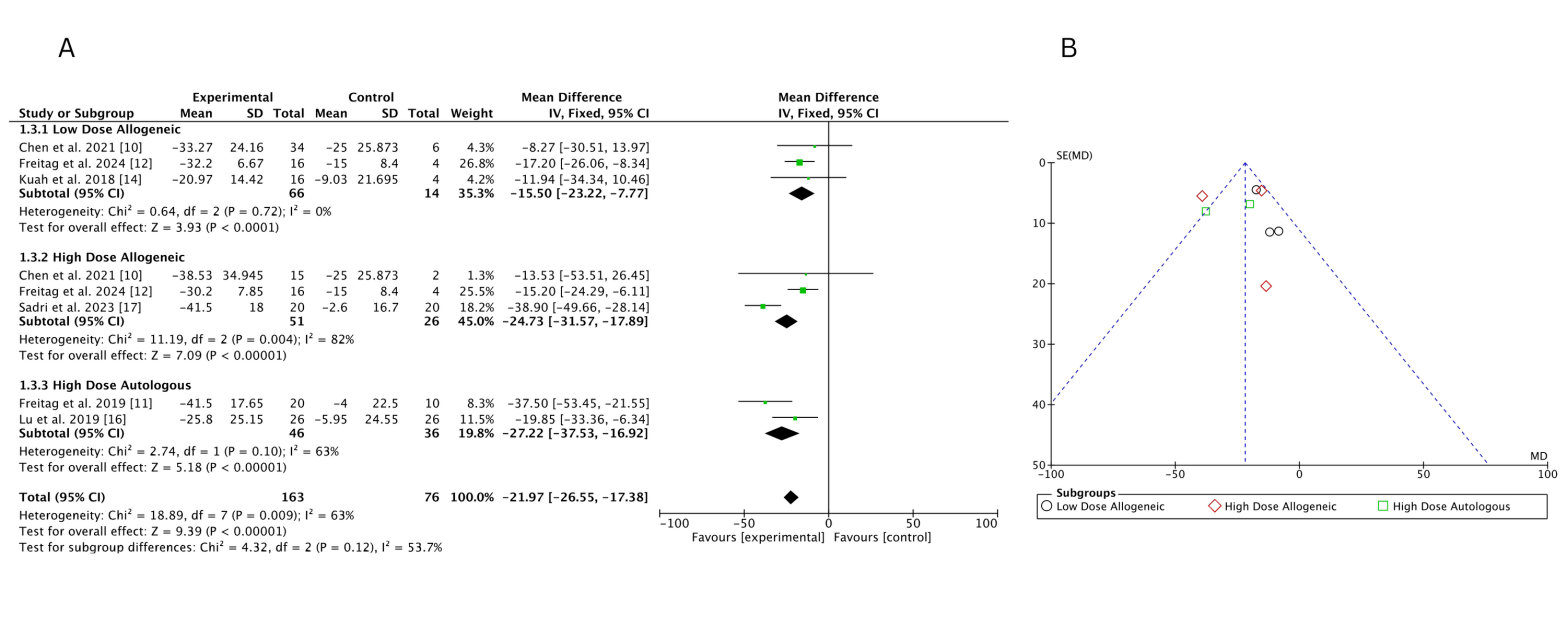
(A) Forest plot of direct comparisons for VAS score change at 12 months; (B) funnel plot assessing publication bias VAS: Visual Analog Scale Sources: [10-12,14,16,17]

High-dose autologous AD-MSCs showed the largest improvement (MD = -27.22; 95% CI: -37.53, -16.92), followed by high-dose allogeneic AD-MSCs (MD = -24.73; 95% CI: -31.57, -17.89). Low-dose allogeneic AD-MSCs demonstrated a smaller but significant effect (MD = -15.50; 95% CI: -23.22, -7.77), with no heterogeneity (I² = 0%). In contrast, heterogeneity was moderate for high-dose autologous AD-MSCs (I² = 63%) and high for high-dose allogeneic AD-MSCs (I² = 82%), indicating some variability among studies, as shown in Figure 16A. The funnel plot, Figure 16B, suggests possible asymmetry for high-dose allogeneic AD-MSCs, indicating potential small-study effects, while other treatment groups appeared more balanced.

Frequentist network meta-analysis: A frequentist NMA was conducted to assess the effectiveness of low-dose allogeneic, high-dose allogeneic, and high-dose autologous AD-MSCs in reducing VAS scores at 12 months. The estimated mean differences (MD) with 95% confidence intervals (CI) for both direct and indirect comparisons are summarized below.

Low-dose allogeneic AD-MSCs showed mixed results, with Freitag et al. (2024) (MD = -16.80; 95% CI: -23.48, -10.12) reporting a notable reduction, whereas Chen et al. (2021) (MD = -8.27; 95% CI: -27.95, 11.41) and Kuah et al. (2018) (MD = -11.94; 95% CI: -34.34, 10.46) had wide confidence intervals, reflecting uncertainty in their effect estimates [10,12,14]. High-dose allogeneic AD-MSCs demonstrated substantial pain reduction, with the strongest effect in Sadri et al. (2023) (MD = -38.90; 95% CI: -49.66, -28.14), followed by Freitag et al. (2024) (MD = -14.80; 95% CI: -21.78, -7.82) [12,17]. However, Chen et al. (2021) (MD = -13.53; 95% CI: -38.72, 11.66) showed wide confidence intervals, indicating variability in treatment response [10]. When comparing high-dose allogeneic to low-dose allogeneic AD-MSCs, the results were inconsistent, with Chen et al. (2021) (MD = -5.26; 95% CI: -24.73, 14.21) and Freitag et al. (2024) (MD = 2.00; 95% CI: -3.05, 7.05) showing overlapping confidence intervals, suggesting no clear superiority [10,12]. High-dose autologous AD-MSCs showed the most pronounced reduction in VAS scores, with Freitag et al. (2019) (MD = -37.50; 95% CI: -53.45, -21.55) and Lu et al. (2019) (MD = -19.84; 95% CI: -33.35, -6.33) confirming significant pain improvement over control [11,16].

**Figure 17:**
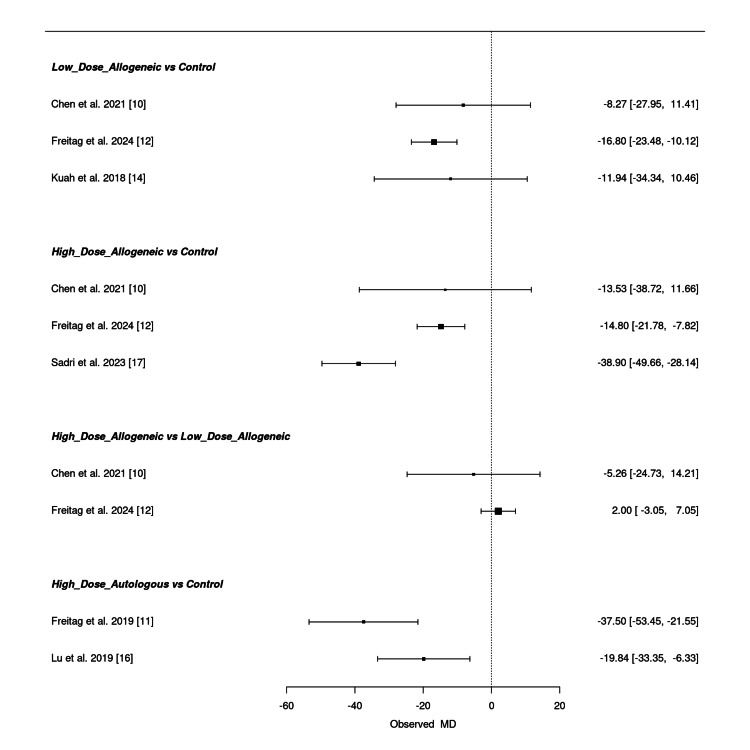
Summary frequentist NMA forest plot for VAS score change at 12 months NMA: network meta-analysis, VAS: Visual Analog Scale Sources: [10-12,14,16,17]

Overall, high-dose autologous AD-MSCs consistently demonstrated the greatest reduction in VAS scores, followed by high-dose allogeneic AD-MSCs, while low-dose allogeneic AD-MSCs exhibited more variable results. Some comparisons had wide confidence intervals, reflecting uncertainty in certain pooled estimates. Figure 17 presents the summary NMA forest plot, visually displaying the relative treatment effects and their corresponding confidence intervals.

SUCRA rankings for VAS score at 12 months: A SUCRA analysis was conducted to rank the effectiveness of low-dose allogeneic, high-dose allogeneic, and high-dose autologous AD-MSCs in reducing VAS scores at 12 months.

**Figure 18:**
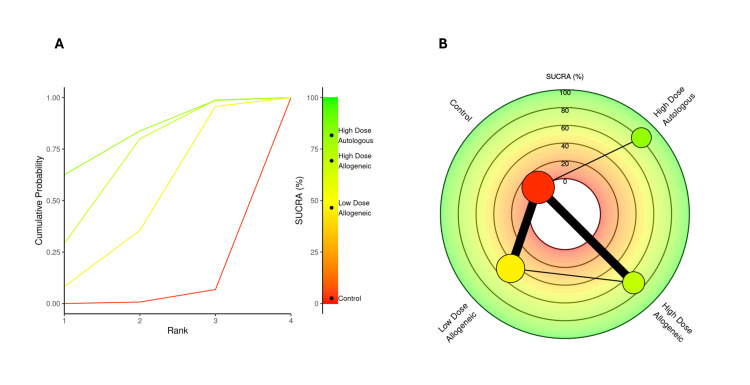
SUCRA rank plots for VAS score at 12 months: (A) radial rank plot – displays cumulative probability ranking for each treatment; (B) Litmus Rank-O-Gram – highlights the relative probability of each treatment being the most effective SUCRA: Surface Under the Cumulative Ranking, VAS: Visual Analog Scale

High-dose autologous AD-MSCs ranked highest (SUCRA: 81.65%), maintaining its position as the most effective treatment across all time points. High-dose allogeneic AD-MSCs ranked second (SUCRA: 69.32%), followed by low-dose allogeneic AD-MSCs (SUCRA: 46.48%). The control group had the lowest SUCRA score (2.55%), confirming its inferiority. The radial rank plot, Figure 18A, illustrates the cumulative probability of each treatment being ranked best, demonstrating that high-dose autologous MSCs continue to show the highest probability of effectiveness. The Litmus Rank-O-Gram, Figure 18B, further highlights the relative ranking probabilities, reinforcing the consistent superiority of high-dose autologous AD-MSCs.

WOMAC Score Change (12 Months)

**Figure 19:**
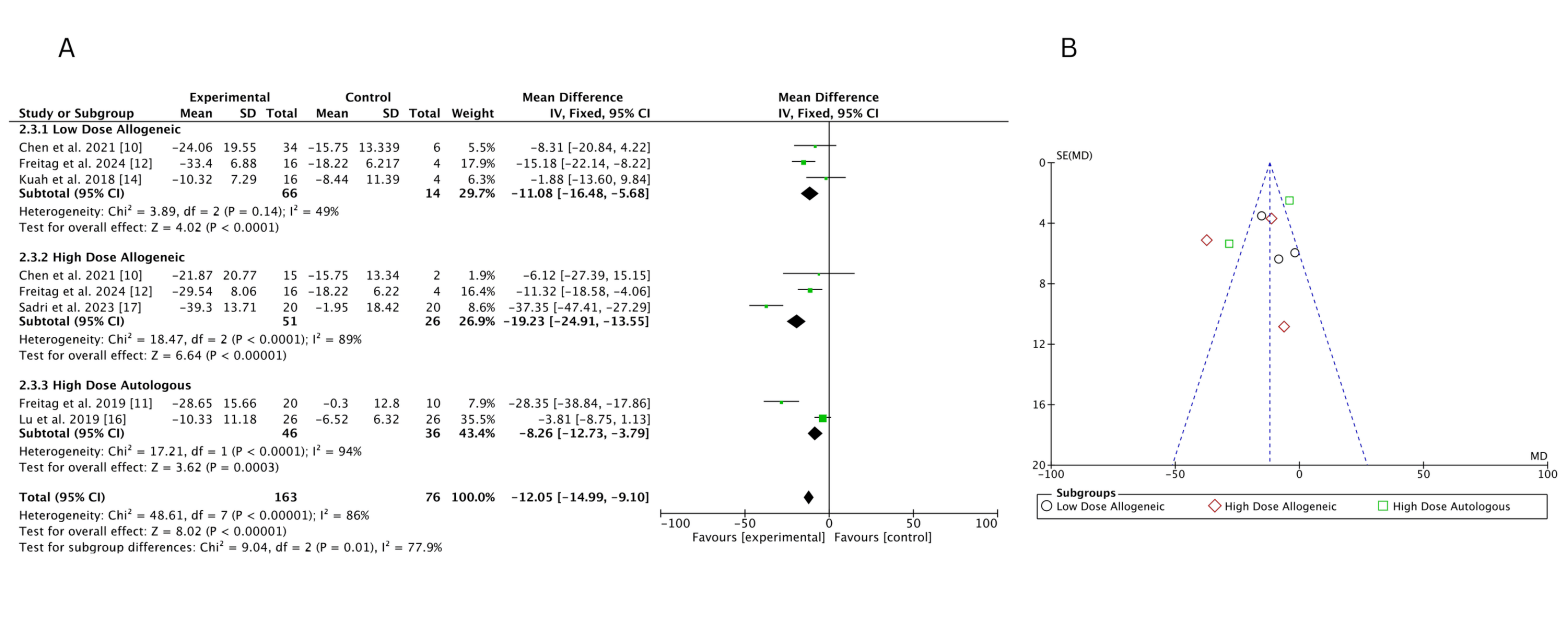
(A) Forest plot of direct comparisons for WOMAC score change at 12 months; (B) funnel plot assessing publication bias WOMAC: Western Ontario and McMaster Universities Osteoarthritis Index Sources: [10-12,14,16,17]

Traditional meta-analysis: At 12 months, a fixed-effects pairwise meta-analysis evaluated WOMAC score changes across low-dose allogeneic, high-dose allogeneic, and high-dose autologous AD-MSCs, with 163 participants included. High-dose allogeneic AD-MSCs showed the greatest improvement (MD = -19.23; 95% CI: -24.91, -13.55), followed by high-dose autologous AD-MSCs (MD = -8.26; 95% CI: -12.73, -3.79). Low-dose allogeneic AD-MSCs had a moderate effect (MD = -11.08; 95% CI: -16.48, -5.68). Heterogeneity was high for both high-dose allogeneic (I² = 89%) and high-dose autologous (I² = 94%) AD-MSCs, while low-dose allogeneic (I² = 49%) showed moderate variability, as shown in Figure 19A. The funnel plot, Figure 19B, suggests some asymmetry for high-dose allogeneic AD-MSCs, raising the possibility of small-study effects but not definitive publication bias.

Frequentist network meta-analysis:* *A frequentist NMA was conducted to assess the effectiveness of low-dose allogeneic, high-dose allogeneic, and high-dose autologous AD-MSCs in reducing WOMAC scores at 12 months. The estimated mean differences (MD) with 95% confidence intervals (CI) for both direct and indirect comparisons are summarized below.

Low-dose allogeneic AD-MSCs showed moderate reductions in WOMAC scores, with Freitag et al. (2024) (MD = -15.18; 95% CI: -20.65, -9.71) showing the largest effect [12]. Chen et al. (2021) (MD = -8.31; 95% CI: -19.65, 3.03) had a wider confidence interval, while Kuah et al. (2018) (MD = -1.88; 95% CI: -13.60, 9.83) showed no significant improvement, reflecting variability in treatment response [10,14]. High-dose allogeneic AD-MSCs demonstrated stronger reductions in WOMAC scores, with Sadri et al. (2023) (MD = -37.35; 95% CI: -47.41, -27.29) showing the most substantial improvement [17]. Freitag et al. (2024) (MD = -11.32; 95% CI: -17.16, -5.48) also reported significant improvement, while Chen et al. (2021) (MD = -6.12; 95% CI: -20.12, 7.88) had a wide confidence interval, indicating uncertainty in its effect estimate [10,12]. When comparing high-dose allogeneic to low-dose allogeneic AD-MSCs, the results were uncertain, with Chen et al. (2021) (MD = 2.19; 95% CI: -10.21, 14.59) and Freitag et al. (2024) (MD = 3.86; 95% CI: -1.33, 9.05) showing overlapping confidence intervals, suggesting no clear superiority between the two treatment groups [10,12]. High-dose autologous AD-MSCs showed the strongest reduction in WOMAC scores, particularly in Freitag et al. (2019) (MD = -28.35; 95% CI: -38.84, -17.86), confirming significant long-term pain relief [11]. However, Lu et al. (2019) (MD = -3.81; 95% CI: -8.75, 1.13) reported a smaller and uncertain effect, with a confidence interval crossing zero [16].

**Figure 20:**
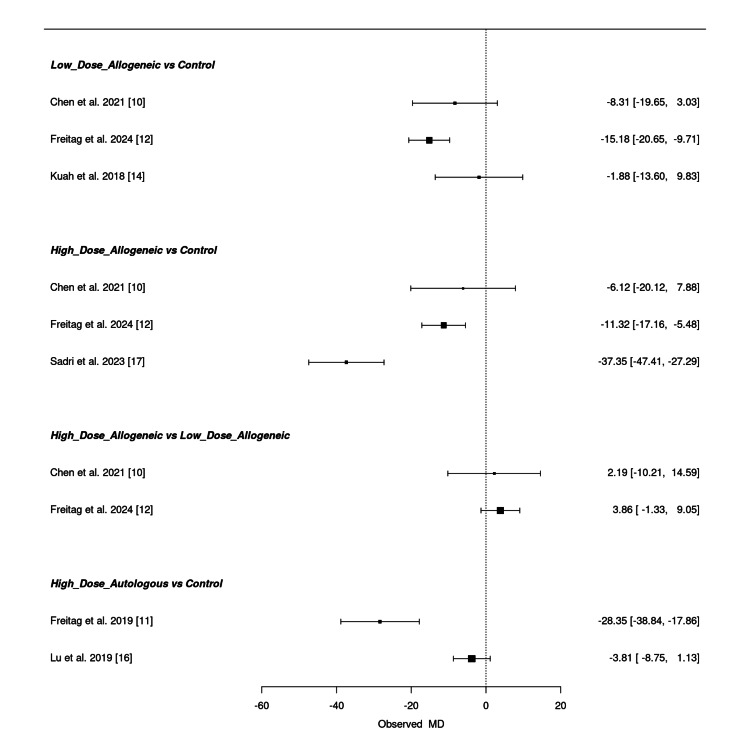
Summary frequentist NMA forest plot for WOMAC score change at 12 months NMA: network meta-analysis, WOMAC: Western Ontario and McMaster Universities Osteoarthritis Index Sources: [10-12,14,16,17]

Overall, high-dose autologous AD-MSCs consistently provided the most significant WOMAC score reductions, followed by high-dose allogeneic AD-MSCs, while low-dose allogeneic AD-MSCs had variable effects. Some comparisons had wide confidence intervals, indicating uncertainty in certain pooled estimates. Figure 20 presents the summary NMA forest plot, visually displaying the relative treatment effects and their corresponding confidence intervals.

SUCRA rankings for WOMAC score at 12 months: A SUCRA analysis was conducted to assess the relative effectiveness of low-dose allogeneic, high-dose allogeneic, and high-dose autologous AD-MSCs in improving WOMAC scores at 12 months.

**Figure 21:**
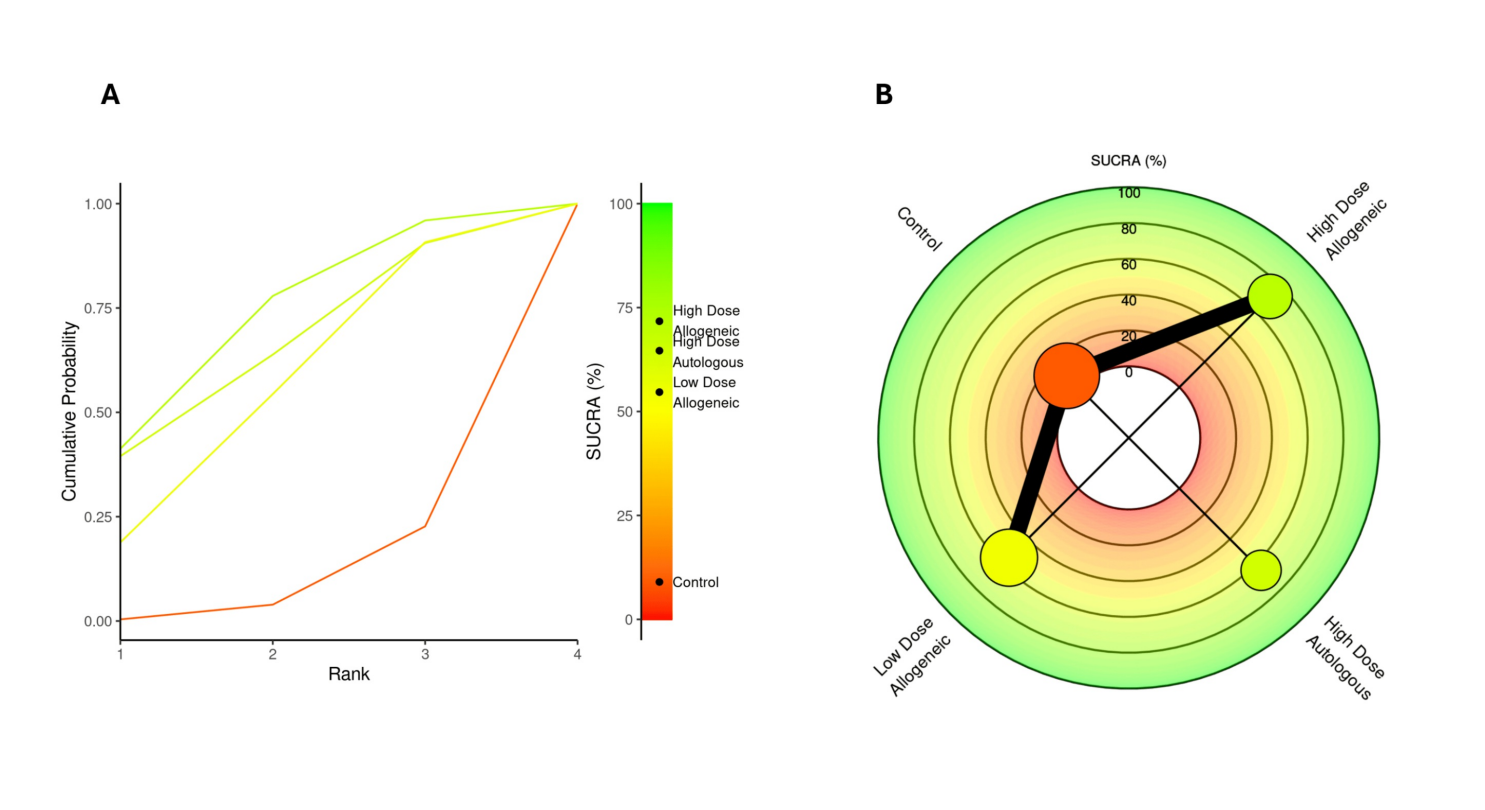
SUCRA rank plots for WOMAC score at 12 months: (A) radial rank plot – displays cumulative probability ranking for each treatment; (B) Litmus Rank-O-Gram – highlights the relative probability of each treatment being the most effective SUCRA: Surface Under the Cumulative Ranking, WOMAC: Western Ontario and McMaster Universities Osteoarthritis Index

At 12 months, high-dose allogeneic AD-MSCs ranked highest (SUCRA: 71.71%), maintaining a slight lead over high-dose autologous AD-MSCs (SUCRA: 64.63%), indicating continued effectiveness in symptom improvement. Low-dose allogeneic AD-MSCs ranked lower (SUCRA: 54.66%), while the control group had the lowest SUCRA score (8.99%), further supporting its limited long-term efficacy. The radial rank plot, Figure 21A, demonstrates the cumulative probability of each treatment being the top-ranked intervention, confirming the dominance of high-dose AD-MSCs treatments. The Litmus Rank-O-Gram, Figure 21B, provides a visual ranking probability distribution, reinforcing the superiority of both high-dose interventions over control and low-dose treatments.

Overall SUCRA Rankings

**Figure 22:**
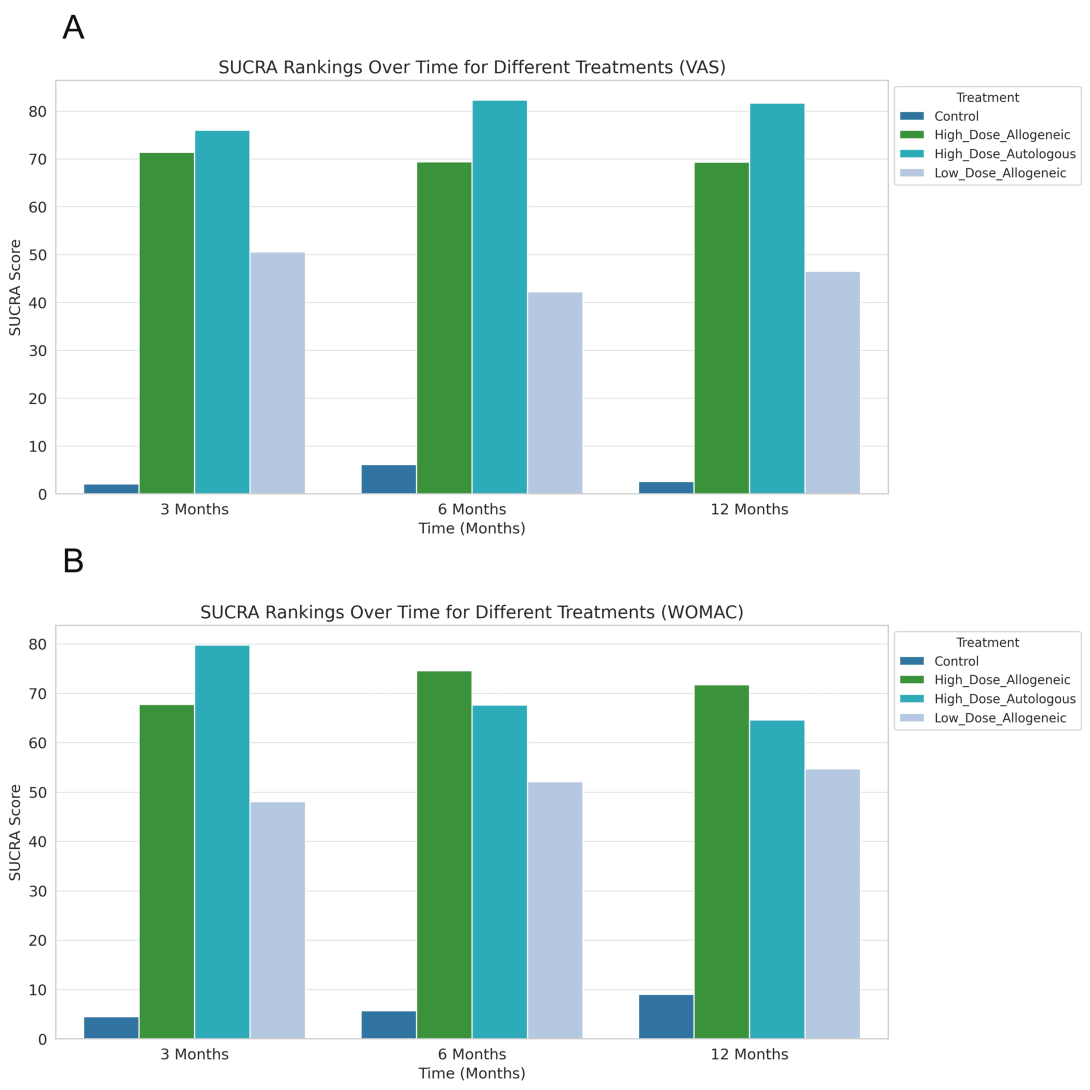
Overall SUCRA rankings across all time points. (A) SUCRA rankings for VAS scores at three, six, and 12 months; (B) SUCRA rankings for WOMAC scores at three, six, and 12 months SUCRA: Surface Under the Cumulative Ranking Curve, VAS: Visual Analog Scale, WOMAC, Western Ontario and McMaster Universities Osteoarthritis Index

The overall SUCRA rankings for pain relief and functional improvement across all time points are summarized in Figure 22.

Adverse Effects (Overall, Across All Time Points)

**Figure 23:**
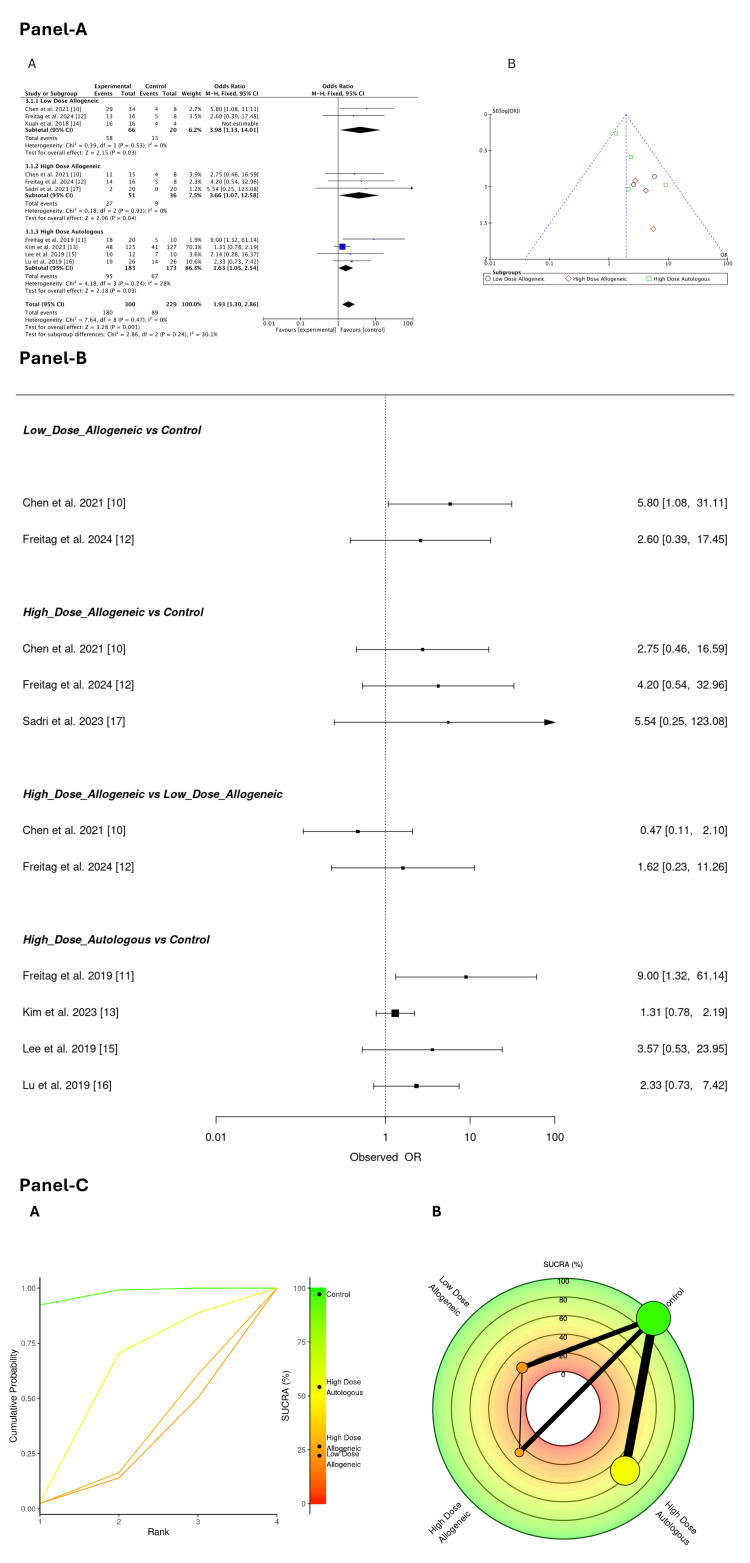
Safety profile of AD-MSC interventions Panel A: (A) Traditional meta-analysis forest plot and (B) funnel plot assessing adverse effects; Panel B: Frequentist network meta-analysis forest plot for adverse effects (overall, across all time points); Panel C: SUCRA ranking plots for adverse effects: (A) radial rank plot and (B) probability rank-o-gram summarizing safety profiles AD-MSC: adipose-derived mesenchymal stem cells, SUCRA: Surface Under the Cumulative Ranking Curve Sources: [10-17]

Figure 23 summarizes the adverse effect outcomes. It presents the traditional meta-analysis forest plot, the frequentist NMA forest plot, and the SUCRA rankings together.

Traditional meta-analysis: A fixed-effects pairwise meta-analysis assessed the risk of adverse effects across low-dose allogeneic, high-dose allogeneic, and high-dose autologous AD-MSCs treatments. A total of 300 participants were analyzed.

Low-dose allogeneic AD-MSCs had the highest odds of adverse effects (OR = 3.98; 95% CI: 1.13, 14.01), followed by high-dose allogeneic AD-MSCs (OR = 3.66; 95% CI: 1.07, 12.58). High-dose autologous AD-MSCs showed a moderate risk (OR = 1.63; 95% CI: 1.05, 2.54). Heterogeneity was low (I² = 0%) for both low-dose and high-dose allogeneic AD-MSCs, indicating consistent findings. However, heterogeneity was moderate for high-dose autologous AD-MSCs (I² = 28%), suggesting some variability among studies, as shown in Figure 23A (A). The funnel plot, Figure 23A (B), showed no strong evidence of publication bias, although some asymmetry was noted for high-dose allogeneic AD-MSCs, suggesting possible small-study effects.

Network meta-analysis: A frequentist NMA was conducted to assess the risk of adverse effects across low-dose allogeneic, high-dose allogeneic, and high-dose autologous AD-MSCs. The estimated odds ratios with 95% confidence intervals for both direct and indirect comparisons are presented in Figure 23B.

Low-dose allogeneic AD-MSCs were associated with an increased risk of adverse effects compared to control, with Chen et al. (2021) (OR = 5.80; 95% CI: 1.08, 31.11) and Freitag et al. (2024) (OR = 2.60; 95% CI: 0.39, 17.45) reporting elevated odds [10,12]. However, confidence intervals were wide, indicating uncertainty in the estimated risk. High-dose allogeneic AD-MSCs also showed an elevated risk of adverse effects, particularly in Sadri et al. (2023) (OR = 5.54; 95% CI: 0.25, 123.08) [17]. Freitag et al. (2024) (OR = 4.20; 95% CI: 0.54, 32.96) and Chen et al. (2021) (OR = 2.75; 95% CI: 0.46, 16.59) reported moderate risks, though confidence intervals were large, reflecting uncertainty in the estimates [10,12]. When comparing high-dose allogeneic to low-dose allogeneic AD-MSCs, the effect was inconclusive, with Chen et al. (2021) (OR = 0.47; 95% CI: 0.11, 2.10) suggesting a potentially lower risk for high-dose allogeneic AD-MSCs, whereas Freitag et al. (2024) (OR = 1.62; 95% CI: 0.23, 11.26) indicated a possible increased risk [10,12]. High-dose autologous AD-MSCs showed mixed risks of adverse effects, with Freitag et al. (2019) (OR = 9.00; 95% CI: 1.32, 61.14) reporting the highest risk [11]. Lee et al. (2019) (OR = 3.57; 95% CI: 0.53, 23.95) and Lu et al. (2019) (OR = 2.33; 95% CI: 0.73, 7.42) reported moderate risks, while Kim et al. (2023) (OR = 1.31; 95% CI: 0.78, 2.19) showed the lowest risk [13,15,16].

Overall, low-dose and high-dose allogeneic AD-MSCs demonstrated an increased risk of adverse effects compared to control, though estimates were imprecise due to wide confidence intervals. High-dose autologous AD-MSCs showed greater variability in adverse effect risks, with some studies indicating a higher likelihood of adverse events.

SUCRA rankings for adverse effects (overall, across all time points): A SUCRA analysis was performed to compare the safety profiles of low-dose allogeneic, high-dose allogeneic, and high-dose autologous AD-MSCs by evaluating the probability of adverse effects occurring over all time points. Figure 23C presents the radial rank plot and Litmus Rank-O-Gram.

Across all time points, the control group ranked highest (SUCRA: 97.16%), indicating the lowest probability of adverse effects. High-dose autologous AD-MSCs followed (SUCRA: 54.08%), suggesting a relatively favorable safety profile. High-dose allogeneic AD-MSCs had a lower SUCRA score (26.52%), and low-dose allogeneic AD-MSCs ranked lowest (SUCRA: 22.24%), suggesting a higher likelihood of adverse events compared to other treatment groups. The radial rank plot, Figure 23C (A), illustrates the cumulative probability of each treatment being the safest option, confirming the superior safety of the control group. The Litmus Rank-O-Gram, Figure 23C (B), visually maps the ranking probabilities, reinforcing the relatively higher safety of high-dose autologous AD-MSCs compared to allogeneic counterparts.

Sensitivity Analyses

Sensitivity analyses (leave-one-out method) were conducted to evaluate the robustness of the meta-analysis findings. For VAS outcomes at three months, sequentially omitting each study resulted in pooled MD values ranging from -12.1 mm (all studies included) to -10.7 mm upon exclusion of Kim et al. (2023), with heterogeneity (I²) reducing from 52% to 35% [13]. At six months, the largest observed shift was 1.2 mm (from -11.3 mm to -10.1 mm), occurring after excluding Freitag et al. (2024); heterogeneity remained largely unaffected (79% vs. 80%) [12]. At 12 months, removing Sadri et al. (2023) produced the largest change, shifting MD from -22.0 mm to -18.2 mm and substantially reducing I² from 63% to 17% [17].

For WOMAC scores, omitting Kim et al. (2023) at three months resulted in an MD shift of 1.5 points (-9.3 to -7.8 on a 0-100 scale), with a corresponding decrease in I² by 37 percentage points [13]. At six months, excluding Lu et al. (2019), it caused a similar effect-size shift of 1.4 points (-12.9 to -11.4), accompanied by a 36-percentage-point reduction in I² [16]. At 12 months, again excluding Lu et al. (2019) led to the largest MD shift (-19.2 to -14.7 points) while excluding Sadri et al. (2023) decreased I² by 13% [16,17].

Importantly, across all time points and outcomes, the omission of any single study did not reverse statistical significance or substantially alter clinical interpretation. Thus, these analyses support the robustness of our pooled estimates.

Consistency Testing Results

To ensure the reliability of the NMA results, we assessed the consistency between direct and indirect estimates using loop inconsistency testing within a frequentist framework and node-splitting analysis within a Bayesian framework [22]. These methods examined whether there were any discrepancies between direct and indirect comparisons in the treatment network for both the VAS and WOMAC scores at 12 months.

Loop inconsistency analysis: The loop inconsistency test compared direct and indirect treatment effects to identify significant deviations in network coherence. 

No statistically significant inconsistency was observed for VAS scores at 12 months, as indicated by p-values > 0.05 across all comparisons. The highest difference between direct and indirect estimates was observed in the Low-Dose Allogeneic AD-MSCs vs. Control comparison (difference: 41.10, 95% CI: -11.81 to 94.02), though it did not reach statistical significance (p = 0.1279). Similarly, comparisons between High-Dose Allogeneic AD-MSCs and Control and High-Dose Autologous AD-MSCs and Control demonstrated no evidence of substantial inconsistency (p = 0.7189 and p = 0.1500, respectively).

For WOMAC scores at 12 months, the loop inconsistency results similarly demonstrated no significant discordance between direct and indirect estimates. The most considerable difference was observed in the High-Dose Allogeneic AD-MSCs vs. Low-Dose Allogeneic AD-MSCs comparison (difference: 39.42, 95% CI: -4.54 to 83.38), but this variation was not statistically significant (p = 0.0788).

Node-splitting analysis: The Bayesian node splitting analysis showed no statistically significant inconsistencies between direct and indirect comparisons. For VAS scores at 12 months, the High-Dose Allogeneic AD-MSCs vs. Low-Dose Allogeneic AD-MSCs comparison had a p-value of 0.2773. At the same time point, the WOMAC scores of the High-Dose Allogeneic AD-MSCs vs. Low-Dose Allogeneic AD-MSCs comparison yielded a p-value of 0.1981, further supporting consistency within the network.

Discussion

Efficacy Overview and Treatment Patterns

Summary of key findings: This study provides a comprehensive evaluation of the efficacy and safety of AD-MSC therapies for knee OA, utilizing both traditional pairwise meta-analysis and network meta-analysis across three, six, and 12-month time points. The findings highlight distinct patterns in pain reduction (VAS scores), functional improvement (WOMAC scores), and adverse effects, allowing for a comparative assessment of treatment options.

Effectiveness of MSC therapies over time: At three months, high-dose autologous AD-MSCs ranked highest for pain relief (VAS SUCRA: 75.99%), followed closely by high-dose allogeneic AD-MSCs (SUCRA: 71.39%), while low-dose allogeneic AD-MSCs ranked significantly lower (SUCRA: 50.53%). A similar trend was observed for functional improvement, where high-dose autologous AD-MSCs (WOMAC SUCRA: 79.75%) and high-dose allogeneic AD-MSCs (SUCRA: 67.72%) outperformed low-dose AD-MSCs therapy (SUCRA: 48.06%).

At six months, high-dose autologous AD-MSCs remained the most effective for pain reduction (VAS SUCRA: 82.27%), but the gap between autologous and allogeneic high-dose AD-MSCs narrowed. Notably, functional improvement rankings shifted, with high-dose allogeneic MSCs surpassing autologous AD-MSCs for WOMAC improvement (SUCRA: 74.59% vs. 67.62%).

At 12 months, high-dose autologous AD-MSCs continued to provide the highest pain relief (VAS SUCRA: 81.65%), while high-dose allogeneic AD-MSCs ranked highest for functional improvement (WOMAC SUCRA: 71.71%). Across all time points, the control group consistently ranked lowest, confirming its limited efficacy in managing OA symptoms compared to AD-MSC-based therapies.

VAS vs. WOMAC trends (alignment and divergence): The treatment rankings for pain relief (VAS) and functional improvement (WOMAC) showed both alignment and divergence over time. At three months, high-dose autologous AD-MSCs ranked highest for both outcomes, indicating a strong early effect on both symptom relief and joint function. However, as time progressed, the rankings began to diverge. At six months, high-dose autologous AD-MSCs remained the most effective for pain reduction (VAS SUCRA: 82.27%), while high-dose allogeneic AD-MSCs emerged as the top-ranked treatment for functional improvement (WOMAC SUCRA: 74.59%). This divergence persisted at 12 months, with high-dose autologous AD-MSCs maintaining their leading position for pain relief (VAS SUCRA: 81.65%) and high-dose allogeneic AD-MSCs continuing to rank highest for functional recovery (WOMAC SUCRA: 71.71%).

These trends suggest that distinct biological mechanisms may underlie short-term pain relief versus longer-term improvements in joint function. Autologous AD-MSCs may exert their early analgesic effects through rapid immune modulation, high cytokine secretion, and prompt suppression of inflammation. In contrast, allogeneic AD-MSCs may provide delayed but durable benefits by promoting sustained tissue remodeling and gradual joint structure and function improvements.

Short-term vs. long-term efficacy*: *The findings support a two-phase model of AD-MSC therapy. High-dose autologous AD-MSCs consistently delivered the greatest pain relief (VAS) across all time points three, six, and 12 months, suggesting strong and sustained anti-inflammatory effects. In contrast, high-dose allogeneic AD-MSCs demonstrated superior outcomes in functional improvement (WOMAC) at both six and 12 months, indicating a delayed but durable impact on joint recovery. This highlights the differential therapeutic mechanisms of autologous and allogeneic AD-MSCs, where autologous AD-MSCs primarily drive sustained inflammation suppression and symptom relief, whereas allogeneic AD-MSCs enhance long-term joint recovery through prolonged immune modulation and cartilage repair [5,23,24].

Dose-dependent responses (high vs. low-dose AD-MSCs): A clear dose-dependent response was observed, with high-dose AD-MSCs consistently improving pain relief and functional outcomes across all time points. This pattern supports the hypothesis that higher concentrations of AD-MSCs enhance therapeutic efficacy, a finding also supported by previous network meta-analyses [25].

While low-dose AD-MSCs conferred some clinical benefit, their effects were modest compared to high-dose treatments. These results suggest that optimizing AD-MSC dosage plays a critical role in maximizing therapeutic outcomes, particularly in patients with more advanced or symptomatic osteoarthritis.

Biological Insights and Ranking Validity

Biological mechanisms underlying source-dependent effects: The differing efficacy patterns of autologous and allogeneic AD-MSCs over time may be attributed to variations in cell survival, immunogenicity, and mechanisms of action. High-dose autologous AD-MSCs consistently provided the greatest pain relief at three, six, and 12 months, likely due to rapid cell integration, potent early paracrine signaling, and strong anti-inflammatory cytokine release, particularly IL-10 and TGF-β [2, 3]. Autologous stem cells are patient-derived, so they are less likely to be cleared by the immune system, allowing for more robust early paracrine signaling and rapid symptom relief.

In contrast, while allogeneic AD-MSCs are not self-derived, they exhibit immune-privileged properties and can modulate host immunity. These effects, including inhibition of T-cell proliferation and promoting M2 macrophage polarization, may create a sustained anti-inflammatory and pro-regenerative joint environment [26,27]. This immune reshaping may mitigate rejection and enable longer-term persistence and integration, supporting cartilage repair and improved function over time [5].

Overall, the observed clinical trends may be explained by the interplay of three primary mechanisms of AD-MSC therapy: (1) paracrine signaling, which mediates early symptom relief through anti-inflammatory cytokines; (2) immune modulation, which alters chronic inflammatory pathways and fosters immune tolerance; and (3) tissue regeneration, which enables longer-term structural repair of joint tissues. These effects evolve dynamically over time and are influenced by AD-MSC source, dose, and biological behavior. This may help explain why autologous cells yield rapid pain relief, whereas allogeneic cells contribute more to long-term functional restoration.

Network consistency and validity of findings: To ensure the reliability of the NMA results, consistency testing was performed using both frequentist loop inconsistency tests and Bayesian node-splitting analysis at 12 months. Neither method identified statistically significant inconsistencies between direct and indirect treatment comparisons, indicating that the assumptions of the network meta-analysis remained valid.

Interpretation of treatment rankings and temporal efficacy trends: The rankings of AD-MSC therapies for pain relief (VAS) and functional improvement (WOMAC) demonstrated notable shifts across time points, reflecting differences in mechanisms of action, dose-dependent responses, and treatment durability. High-dose autologous AD-MSCs provided sustained analgesic effects over time, ranking highest for pain relief at three, six, and 12 months. Notably, high-dose allogeneic AD-MSCs ranked highest for functional improvement at six and 12 months, suggesting a more gradual and sustained regenerative effect on joint function.

Implications of consistency testing on AD-MSC treatment rankings: The consistency testing results confirmed that the NMA upheld its assumptions, with no statistically significant inconsistencies between direct and indirect estimates [22]. Both loop inconsistency testing and Bayesian node-splitting analysis indicated strong agreement across most treatment comparisons, reinforcing confidence in the validity of SUCRA-based treatment rankings.

Despite the network's overall robustness, some comparisons, particularly those involving low-dose allogeneic AD-MSCs, exhibited wider confidence intervals due to limited direct evidence. This suggests that while the general treatment hierarchy remains reliable, rankings for treatments with fewer direct pairwise comparisons should be interpreted with greater caution. Additionally, the absence of long-term follow-up beyond 12 months limits the ability to assess sustained efficacy [28].

Given these findings, the NMA results provide a strong evidence base for guiding treatment selection, but future studies with longer follow-up and additional direct comparisons will be necessary to refine treatment rankings further. 

Safety Considerations

Adverse effects and safety profile: The safety analysis highlighted the important trade-offs between efficacy and adverse effects. Among AD-MSC treatments, low-dose allogeneic AD-MSCs had the highest risk of adverse effects (SUCRA: 22.24%), followed by high-dose allogeneic AD-MSCs (SUCRA: 26.52%). High-dose autologous AD-MSCs, while ranking lower in safety than the control group, remained the safest among AD-MSC therapies (SUCRA: 54.08%). The control group was found to be the safest option (SUCRA: 97.16%).

Serious adverse events (is AD-MSCs therapy risky?): Across the included studies, serious adverse events (SAEs) were rare and occurred at similar or higher rates in control groups compared to AD-MSC treatment groups. No SAE was directly attributed to AD-MSC therapy. For example, one SAE (1.92%) in a control group involved a joint infection requiring study withdrawal [16], while another study [13] reported a pneumonia case in a high-dose autologous AD-MSCs patient and three unrelated SAEs in the control group. Similarly, a third study [10] observed one unrelated SAE in the low-dose allogeneic AD-MSCs group. These findings reinforce the safety of AD-MSC therapy for clinical use in osteoarthritis.

Clinical relevance (are the AEs worth the benefit?): While high-dose AD-MSCs were occasionally associated with transient joint pain or swelling at the injection site, these effects were mild and resolved without intervention. To provide additional clinical context, the most frequently reported adverse events across the included studies included arthralgia, joint swelling, injection site discomfort, and stiffness. In Kim et al. (2023), 48 patients (38.4%) experienced at least one adverse event, with 50 Grade 1 and 22 Grade 2 events and no Grade ≥3 events [13]. Kuah et al. (2018) reported that over 80% of events were mild, with a single severe event (prepatellar bursitis) potentially related to the injection [14]. Lee et al. (2019) documented 22 Grade 1, 9 Grade 2, and 3 Grade 3 events, all resolved without further treatment [15]. Chen et al. (2021) noted treatment-related joint pain (15.8%), arthralgia (14%), and joint swelling (10.5%), with only one Grade ≥3 AE considered related to treatment [10]. These data collectively reinforce the favorable safety profile of AD-MSC therapy, particularly for high-dose autologous preparations. Notably, high-dose autologous AD-MSCs offered the strongest efficacy and ranked safest among all treatment options. In contrast, low-dose allogeneic AD-MSCs had the highest rate of adverse events and provided less clinical benefit, raising questions about their clinical value.

Selecting the appropriate AD-MSC dose and source should be based on individual patient factors such as osteoarthritis severity, symptom burden, and tolerance for mild adverse effects. For patients prioritizing symptom relief and functional recovery, particularly those with moderate to severe disease, high-dose autologous AD-MSCs may offer the most favorable balance of safety and efficacy.

Trade-off between efficacy and safety of AD-MSC therapy: The balance between efficacy and safety is a critical factor in clinical decision-making for AD-MSC therapy in knee osteoarthritis. High-dose AD-MSCs demonstrated the greatest improvements in both pain relief (VAS) and functional outcomes (WOMAC), particularly when autologous AD-MSCs were used. Importantly, adverse event risk did not increase uniformly with dose. High-dose autologous AD-MSCs exhibited the most favorable safety profile, while low-dose allogeneic AD-MSCs had the highest incidence of mild-to-moderate adverse events. These findings challenge the assumption that lower doses are inherently safer and highlight the importance of considering both cell source and dose when evaluating AD-MSC therapies.

Clinical Translation and Future Directions

Limitations and clinical implications: Several limitations must be acknowledged. Most included studies received partial or complete industry sponsorship, which may introduce publication or performance bias. Additionally, relatively few direct head-to-head comparisons exist between dose levels or MSC sources, requiring reliance on indirect evidence within the NMA framework. These limitations restrict generalizability and underscore the need for standardized outcome definitions, longer follow-up, and non-industry-funded trials.

A further notable notable source of heterogeneity in this meta-analysis is differences in outcome measurement tools and scale reporting. While all included studies assessed pain and function, the specific implementation of these measures varied. Some studies utilized a 0-100 mm VAS, while others used a 0-10 Numeric Pain Rating Scale (NPRS), introducing potential scale-related inconsistencies. Similarly, WOMAC scores were reported either as raw totals (maximum 96), normalized percentages (0-100), or as subscales without a unified format. Although we applied standardized conversions to harmonize these scales, such differences in measurement and reporting may have contributed to the observed between-study heterogeneity. However, our sensitivity analysis demonstrated that our findings were not dependent on any single study; despite moderate shifts in effect size and heterogeneity when removing influential studies, the findings' overall significance and direction remained robust. This enhances confidence in the reliability of our pooled estimates. These inconsistencies highlight the importance of future research adopting unified reporting standards to facilitate more accurate comparisons.

From a clinical perspective, treatment selection may based on practical and biological factors. Autologous AD-MSCs may be ideal for younger patients with early to moderate OA (Kellgren-Lawrence Grade II-III), given the higher regenerative capacity of autologous cells and their safety profile. However, autologous therapy involves liposuction, cell processing, and delayed treatment, which may be costly and logistically intensive. Allogeneic AD-MSCs, by contrast, offer off-the-shelf availability, making them more feasible for older patients, patients with comorbidities, or those seeking faster access to therapy. Depending on patient characteristics and resource availability, clinicians may consider a phased strategy, using autologous MSCs for early intervention and allogeneic products for sustained joint support.

Implications for clinical decision-making: The absence of treatment-related serious adverse events strengthens AD-MSC therapy's overall safety profile, supporting its clinical viability. Clinicians should inform patients about the possibility of mild, self-limiting side effects while reassuring them that serious complications are rare [24,29].

These findings suggest that high-dose AD-MSC therapy, particularly autologous AD-MSCs, may offer a favorable balance of safety and efficacy for many patients. However, the choice between autologous and allogeneic sources should be individualized based on patient goals, tolerability, and whether rapid symptom relief or long-term functional improvement is prioritized.

Therapeutic role of AD-MSCs in knee OA: The findings of this network meta-analysis provide valuable insights into the clinical application of AD-MSC therapy for knee osteoarthritis. High-dose AD-MSCs, particularly autologous AD-MSCs, demonstrated superior efficacy in pain relief (VAS), while high-dose allogeneic AD-MSCs showed greater improvements in long-term joint function (WOMAC). These findings suggest that AD-MSC therapy may offer a more effective alternative to conventional intra-articular treatments such as corticosteroids or hyaluronic acid, especially for patients who have not responded to traditional pharmacologic or injection-based therapies [27,30].

While surgical intervention such as total knee replacement (TKR) remains the definitive option for advanced knee OA, AD-MSC therapy presents a promising regenerative approach for patients aiming to delay or avoid surgery [1]. One study [31] even reported that patients treated with MSCs in one knee and TKA in the other experienced better functional outcomes and fewer complications in the MSC-treated knee. Although these findings are preliminary, they underscore MSC therapy’s potential role as an intermediate option between conservative care and surgery.

Given its favorable safety and efficacy profile, AD-MSC therapy may serve as a valuable treatment strategy for patients with moderate to severe OA who are not yet candidates for surgery. Individualized treatment decisions should consider patient preferences, disease severity, and the desire to delay surgical intervention.

Should clinicians prefer autologous or allogeneic MSCs?: The choice between autologous and allogeneic AD-MSCs should be guided by treatment goals and clinical context. High-dose autologous AD-MSCs demonstrated stronger and more consistent pain relief across all time points, making them a compelling option for patients seeking rapid symptom control. In contrast, high-dose allogeneic AD-MSCs ranked higher for functional improvement at six and 12 months, suggesting greater long-term regenerative effects. Practical considerations also influence clinical decision-making. Autologous AD-MSCs require harvesting and processing from the patient, which may increase procedural burden, cost, and treatment delay. Meanwhile, allogeneic AD-MSCs offer off-the-shelf convenience, enabling easier access and potentially broader scalability [7].

Future research priorities: While this study provides strong support for the clinical use of AD-MSC therapy in knee osteoarthritis, several key research gaps remain. One major limitation is the short follow-up duration in most studies. Although this network meta-analysis assessed outcomes up to 12 months, the long-term durability of AD-MSC therapy remains uncertain. Future trials should extend follow-up beyond 24 months to determine whether improvements in pain and function are sustained or decline over time [28]. Long-term safety monitoring will also be critical to assess any delayed adverse effects, including immune responses or intra-articular complications such as fibrosis.

Another important priority is the need for direct, head-to-head randomized controlled trials comparing autologous and allogeneic AD-MSC. While this NMA provided indirect comparisons, direct evidence is needed to clarify differences in clinical efficacy, immune compatibility, and persistence within the joint environment [25,31].

Additionally, future studies should explore biomarker-based patient selection strategies to optimize AD-MSC therapy. Given the heterogeneity of OA progression and treatment response, identifying biological predictors of success could improve outcomes and reduce unnecessary exposure for non-responders. Biomarkers such as IL-6, TNF-α, synovial fluid composition, or cartilage degradation products may help identify patients most likely to benefit from AD-MSC therapy [32,33]. This personalized approach could enhance treatment precision and maximize the clinical value of AD-MSC in OA management.

## Conclusions

This network meta-analysis demonstrates that high-dose AD-MSC therapy offers superior pain relief and functional improvement compared to conventional intra-articular treatments for knee osteoarthritis. A clear dose-dependent response was observed, with higher doses consistently outperforming lower doses in symptom relief and functional outcomes. High-dose autologous AD-MSCs were most effective for rapid and sustained pain reduction, while high-dose allogeneic AD-MSCs ranked highest for long-term functional recovery at six and 12 months, suggesting distinct therapeutic profiles. Although mild to moderate adverse effects such as transient joint pain and swelling were more common in high-dose groups, serious adverse events were rare and occurred at similar rates to control groups, supporting the overall safety of AD-MSC therapy.

Clinically, high-dose autologous AD-MSCs may be preferred for patients seeking immediate symptom relief, whereas high-dose allogeneic AD-MSCs may better support long-term joint function. Given its favorable safety profile and comparative effectiveness, AD-MSC therapy represents a promising alternative for patients who have not responded to conventional treatments such as corticosteroids or hyaluronic acid. Future research should prioritize long-term follow-up beyond 12 months, direct head-to-head comparisons of autologous and allogeneic AD-MSCs, biomarker-based patient selection, and the optimization of dosing protocols. This study provides a framework to guide dose- and source-specific selection of AD-MSC therapies in the management of knee osteoarthritis.

## References

[1] Vohra M, Arora SK (2023). Mesenchymal stem cells—the master immunomodulators. Explor Immunol.

[2] Weiss AR, Dahlke MH (2019). Immunomodulation by mesenchymal stem cells (MSCs): mechanisms of action of living, apoptotic, and dead MSCs. Front Immunol.

[3] Strioga M, Viswanathan S, Darinskas A, Slaby O, Michalek J (2012). Same or not the same? Comparison of adipose tissue-derived versus bone marrow-derived mesenchymal stem and stromal cells. Stem Cells Dev.

[4] Khaboushan AS, Ebadpour N, Moghadam MM, Rezaee Z, Kajbafzadeh AM, Zolbin MM (2024). Cell therapy for retinal degenerative disorders: a systematic review and three-level meta-analysis. J Transl Med.

[5] Lopa S, Colombini A, Moretti M, de Girolamo L (2019). Injective mesenchymal stem cell-based treatments for knee osteoarthritis: from mechanisms of action to current clinical evidences. Knee Surg Sports Traumatol Arthrosc.

[6] Faraji N, Ebadpour N, Abavisani M, Gorji A (2025). Unlocking hope: therapeutic advances and approaches in modulating the Wnt pathway for neurodegenerative diseases. Mol Neurobiol.

[7] Pers YM, Rackwitz L, Ferreira R (2016). Adipose mesenchymal stromal cell-based therapy for severe osteoarthritis of the knee: a phase I dose-escalation trial. Stem Cells Transl Med.

[8] Wang Y, Chen X, Cao W, Shi Y (2014). Plasticity of mesenchymal stem cells in immunomodulation: pathological and therapeutic implications. Nat Immunol.

[9] Page MJ, McKenzie JE, Bossuyt PM (2021). The PRISMA 2020 statement: an updated guideline for reporting systematic reviews. BMJ.

[10] Chen CF, Hu CC, Wu CT (2021). Treatment of knee osteoarthritis with intra-articular injection of allogeneic adipose-derived stem cells (ADSCs) ELIXCYTE®: a phase I/II, randomized, active-control, single-blind, multiple-center clinical trial. Stem Cell Res Ther.

[11] Freitag J, Bates D, Wickham J (2019). Adipose-derived mesenchymal stem cell therapy in the treatment of knee osteoarthritis: a randomized controlled trial. Regen Med.

[12] Freitag J, Chamberlain M, Wickham J, Shah K, Cicuttini F, Wang Y, Solterbeck A (2024). Safety and efficacy of an allogeneic adipose-derived mesenchymal stem cell preparation in the treatment of knee osteoarthritis: a phase I/IIa randomised controlled trial. Osteoarthr Cartil Open.

[13] Kim KI, Lee MC, Lee JH (2023). Clinical efficacy and safety of the intra-articular injection of autologous adipose-derived mesenchymal stem cells for knee osteoarthritis: a phase III, randomized, double-blind, placebo-controlled trial. Am J Sports Med.

[14] Kuah D, Sivell S, Longworth T (2018). Safety, tolerability and efficacy of intra-articular Progenza in knee osteoarthritis: a randomized double-blind placebo-controlled single ascending dose study. J Transl Med.

[15] Lee WS, Kim HJ, Kim KI, Kim GB, Jin W (2019). Intra-articular injection of autologous adipose tissue-derived mesenchymal stem cells for the treatment of knee osteoarthritis: a phase IIb, randomized, placebo-controlled clinical trial. Stem Cells Transl Med.

[16] Lu L, Dai C, Zhang Z (2019). Treatment of knee osteoarthritis with intra-articular injection of autologous adipose-derived mesenchymal progenitor cells: a prospective, randomized, double-blind, active-controlled, phase IIb clinical trial. Stem Cell Res Ther.

[17] Sadri B, Hassanzadeh M, Bagherifard A (2023). Cartilage regeneration and inflammation modulation in knee osteoarthritis following injection of allogeneic adipose-derived mesenchymal stromal cells: a phase II, triple-blinded, placebo controlled, randomized trial. Stem Cell Res Ther.

[18] Sterne JA, Savović J, Page MJ (2019). RoB 2: a revised tool for assessing risk of bias in randomised trials. BMJ.

[19] McGuinness LA, Higgins JP (2021). Risk-of-bias VISualization (robvis): an R package and Shiny web app for visualizing risk-of-bias assessments. Res Synth Methods.

[20] Nevill CR, Cooper NJ, Sutton AJ (2023). A multifaceted graphical display, including treatment ranking, was developed to aid interpretation of network meta-analysis. J Clin Epidemiol.

[21] Owen RK, Bradbury N, Xin Y, Cooper N, Sutton A (2019). MetaInsight: An interactive web-based tool for analyzing, interrogating, and visualizing network meta-analyses using R-shiny and netmeta. Res Synth Methods.

[22] Higgins JP, Jackson D, Barrett JK, Lu G, Ades AE, White IR (2012). Consistency and inconsistency in network meta-analysis: concepts and models for multi-arm studies. Res Synth Methods.

[23] Chen MC, Chang JP, Chang TH (2015). Unraveling regulatory mechanisms of atrial remodeling of mitral regurgitation pigs by gene expression profiling analysis: role of type I angiotensin II receptor antagonist. Transl Res.

[24] Lamo-Espinosa JM, Mora G, Blanco JF (2016). Intra-articular injection of two different doses of autologous bone marrow mesenchymal stem cells versus hyaluronic acid in the treatment of knee osteoarthritis: multicenter randomized controlled clinical trial (phase I/II). J Transl Med.

[25] Huang Z, Zhang S, Cao M (2023). What is the optimal dose of adipose-derived mesenchymal stem cells treatment for knee osteoarthritis? A conventional and network meta-analysis of randomized controlled trials. Stem Cell Res Ther.

[26] Aggarwal S, Pittenger MF (2005). Human mesenchymal stem cells modulate allogeneic immune cell responses. Blood.

[27] Jin WS, Yin LX, Sun HQ, Zhao Z, Yan XF (2025). Mesenchymal stem cells injection is more effective than hyaluronic acid injection in the treatment of knee osteoarthritis with similar safety: a systematic review and meta-analysis. Arthroscopy.

[28] Davatchi F, Sadeghi Abdollahi B, Mohyeddin M, Nikbin B (2016). Mesenchymal stem cell therapy for knee osteoarthritis: 5 years follow-up of three patients. Int J Rheum Dis.

[29] Wiggers TG, Winters M, Van den Boom NA, Haisma HJ, Moen MH (2021). Autologous stem cell therapy in knee osteoarthritis: a systematic review of randomised controlled trials. Br J Sports Med.

[30] Kolasinski SL, Neogi T, Hochberg MC (2020). 2019 American College of Rheumatology/Arthritis Foundation guideline for the management of osteoarthritis of the hand, hip, and knee. Arthritis Rheumatol.

[31] Lin J, Huang J, Jiao Z (2025). Mesenchymal stem cells for osteoarthritis: recent advances in related cell therapy. Bioeng Transl Med.

[32] Choi Y, Shin S, Son HJ (2023). Identification of potential biomarkers related to mesenchymal stem cell response in patients with Alzheimer's disease. Stem Cell Res Ther.

[33] Chahal J, Gómez-Aristizábal A, Shestopaloff K (2019). Bone marrow mesenchymal stromal cell treatment in patients with osteoarthritis results in overall improvement in pain and symptoms and reduces synovial inflammation. Stem Cells Transl Med.

